# Exploring the parameters of central redox hub for screening salinity tolerant rice landraces of coastal Bangladesh

**DOI:** 10.1038/s41598-022-17078-2

**Published:** 2022-07-29

**Authors:** Uthpal Krishna Roy, Soumen Bhattacharjee

**Affiliations:** 1 Plant Physiology and Biochemistry Research Laboratory, Department of Botany, UGC Centre for Advanced Study, The University of Burdwan, Burdwan, West Bengal 713104 India; 2 Plant Physiology and Biochemistry Research Laboratory, Department of Botany, UGC Centre for Advanced Study, The University of Burdwan, Burdwan, West Bengal 713104 India; 3 Present Address: Department of Botany, University of Rajshahi, Rajshahi, 6205 Bangladesh

**Keywords:** Biochemistry, Plant sciences

## Abstract

Regulation of oxidative stress towards origin of favorable internal redox cue plays a decisive role in salinity stress acclimation and least studied in rice and hence is the subject of present investigation. Redox landscaping of seedlings of ten experimental land races of rice of coastal Bangladesh grown under post imbibitional salinity stress (PISS) has been done through characterization of ROS-antioxidant interaction dynamics at metabolic interface, transcriptional reprogramming of redox-regulatory genes along with the assessment of biomarkers of oxidative threat for standardizing redox strategies and quality parameters for screening. The results exhibited a strong correlation between salinity induced redox status (pro-oxidant/antioxidant ratio, efficacy of H_2_O_2_ turnover through integrated RboH-Ascorbate–Glutathione/Catalase pathway and estimation of sensitive redox biomarkers of oxidative deterioration) and germination phenotypes of all landraces of rice. Transcript abundance of the marker genes of the enzymes associated with central antioxidant hub for H_2_O_2_ processing (*CatA*, *OsAPx2, SodCc2, GRase* and *RboH*) of all experimental landraces of the rice advocate the central role of H_2_O_2_ turnover dynamics in regulating redox status and salinity tolerance. Landraces suffering greater loss of abilities of decisive regulation of H_2_O_2_ turnover dynamics exhibited threat on the oxidative windows of the germinating seeds under salinity.

## Introduction

Soil salinization depending on its magnitude and duration of exposure induce ion and osmotic stresses which if remains unabated instigate oxidative threat and hamper productivity of crops including rice^1–5^. Plant explores integrated redox hub to combat salinity and restore redox homeostasis of the system for prevention of salinity induced oxidative threat^6–10^. The redox-state of tissue which largely depends on antioxidant couples such as ascorbate/dehydroascorbate (AsA/DHA) and glutathione/glutathione disulfide (GSH/GSSG) determine the fate of environmentally challenged plants including salinity^11,12^. These couples otherwise known as “heart of the redox hub” which plant explore largely for the redox-regulations that enables them to convert the oxidative threat into redox signaling. The innately greater abundance of ascorbate (AsA) and reduced glutathione (GSH) along with their oxidized forms in cellular system make them the most sensitive and efficient redox couples associated with redox buffering under stress^8,13^. However, when the interconnection between these two redox couples were established, it was found that the electrons from reducing equivalent NADPH + H^+^ can flow through GSH and AsA ultimately reducing hydrogen peroxide (H_2_O_2_) with the involvement of the enzymes ascorbate peroxidase (APOX) [EC 1.11.1.11], monodehydroascorbate reductase (MDHAR) [EC 1.6.5.4], dehydroascorbate reductase (DHAR) [EC 1.8.5.1], and glutathione reductase (GR) [EC 1.8.1.7]^8,12,14^.

Salinity stress in rice, in general modulate the endogenous level of ascorbate and glutathione as well as enhance the activities of other important antioxidative defense enzymes associated with ascorbate—glutathione pathway to combat the oxidative threat like lipid peroxidation, protein oxidation etc^7,8,15–17^. Several workers have observed close relationship between antioxidative defense mechanism and salinity stress responsiveness^16–18^. In this regard the importance of ascorbate–glutathione (A-G) system as an important redox strategy for maintenance of the redox-homeostasis of salinity stress in crops is gaining importance in the back drop of emmerging concept of redox signaling^12,19–21^. Further, the extremely complicated nature of salinity stress tolerance mechanism with ROS-antioxidant interaction at metabolic interface as a central mechanism associated with the formation of internal redox cue leads to our incapability to improve our understanding of salt tolerance. Most importantly little attention has been paid to understand original internal redox-cue through antioxidant buffering involving A-G system as key component of redox regulation of rice necessary for salinity tolerance. In this regard reliable redox-biomarkers and the competence of A-G pathway (in terms of transcriptional regulation of genes of the enzymes involved in the activities as well as redox turnover of ascorbate and glutathione) may be used as salinity stress tolerance markers^12,21–23^. Finally, the integration of molecular data with these redox-metabolic data and physiological phenotyping will assist the workers not only to understand the genetic regulation of salinity tolerance and their response but also can be successfully implemented for screening salinity tolerant crops^8,12,24^. In this backdrop, the objective of the present study was framed to screen salinity tolerant landraces of coastal areas of Bangladesh capable of fine tuning central redox hub (integrated RboH-Ascorbate–Glutathione/Catalase pathway) at the metabolic interface for the mitigation of oxidative threats and regulating generation of endogenous redox cue.

## Results

### Redox strategies involving integrated Rboh-ascorbate–glutathione/catalase pathway in PISS-raised seedling of ten rice landraces of coastal areas of Sundarban, Bangladesh

**Figure 1:**
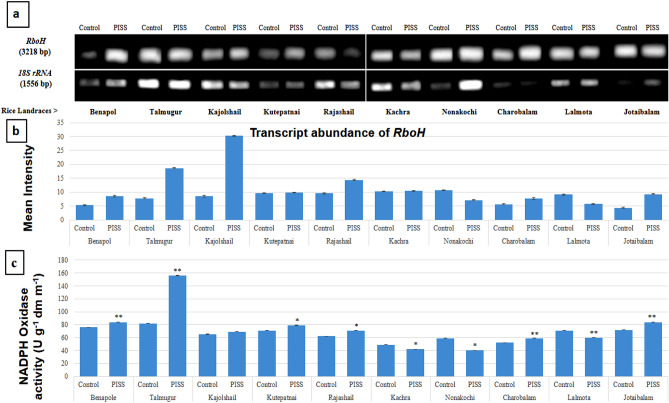
Comparative transcript abundance of gene *RboH* in PISS (200 mM NaCl) raised seedling of ten experimental land races of rice of Sundarban, Bangladesh (**a**). Semi – quantitative RT–PCR was performed as described in materials and methods. The rice 18S rRNA control reaction below each set of corresponding gene reactions were conducted on equivalent cDNA batches to verify equivalent loading reaction volumes on the gel. Antioxidant genes were amplified for 25 cycles. Borders in the gel images represent the areas in which they are juxtaposed from two different gels. (**b**) Bar diagram represents mean intensity of relative expression of genes. (**c**) Activity of the corresponding enzyme RboH of PISS (200 mM NaCl) raised seedling of ten experimental land races of rice.

**Figure 2:**
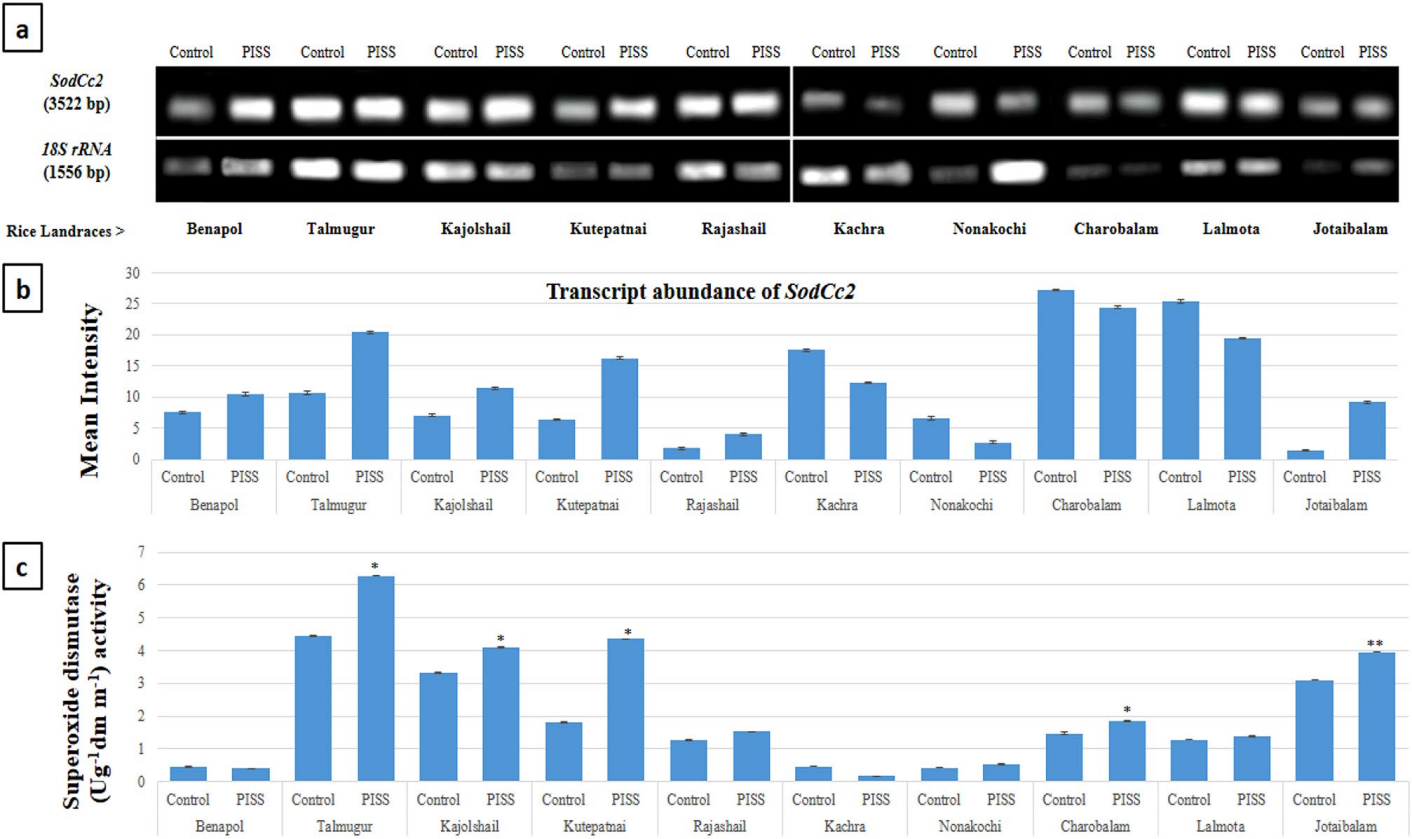
Comparative transcript abundance of the gene *Sod*Cc2 from untreated control and PISS (200 mM NaCl) raised seedling of ten experimental land races of rice of Sundarban, Bangladesh (**a**). Semi – quantitative RT–PCR was performed as described in materials and methods. The rice 18S rRNA control reaction below each set of corresponding gene reactions were conducted on equivalent cDNA batches to verify equivalent loading reaction volumes on the gel. Antioxidant genes were amplified for 25 cycles. Borders in the gel images represent the areas in which they are juxtaposed from two different gels. (**b**) Bar diagram represents mean intensity of relative expression of genes. (**c**) Activity of the corresponding enzyme SOD of PISS (200 mM NaCl) raised seedling of ten experimental land races of rice.

**Figure 3:**
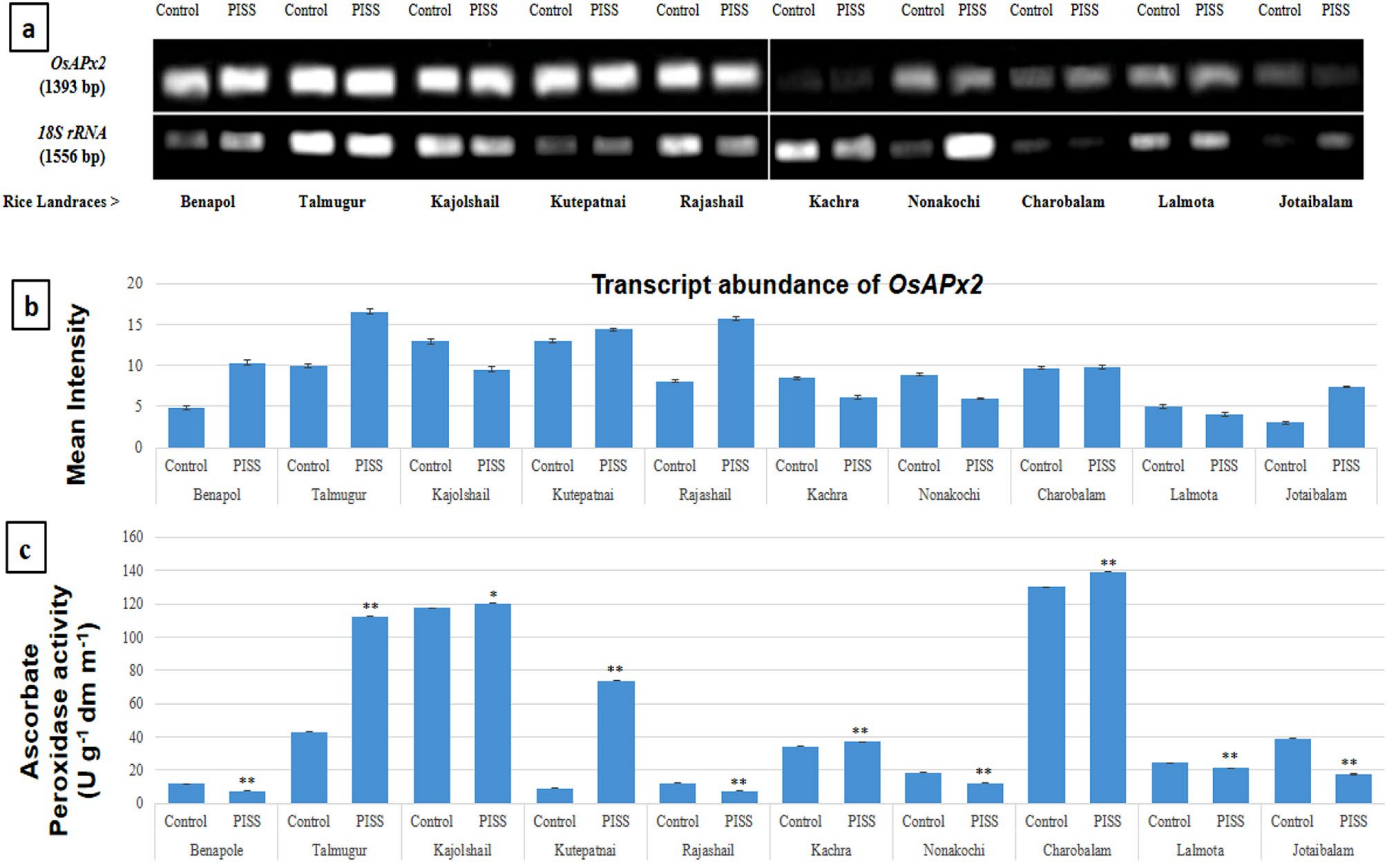
Comparative transcript abundance of gene *OsAPx2* from untreated control and PISS (200 mM NaCl) raised seedling of ten experimental land races of rice of Sundarban, Bangladesh (**a**). Semi – quantitative RT–PCR was performed as described in materials and methods. The rice 18S rRNA control reaction below each set of corresponding gene reactions were conducted on equivalent cDNA batches to verify equivalent loading reaction volumes on the gel. Antioxidant genes were amplified for 25 cycles. Borders in the gel images represent the areas in which they are juxtaposed from two different gels. (**b**) Bar diagram represents mean intensity of relative expression of genes. (**c**) Activity of the corresponding enzyme ascorbate peroxidase of PISS (200 mM NaCl) raised seedling of ten experimental land races of rice.

**Figure 4:**
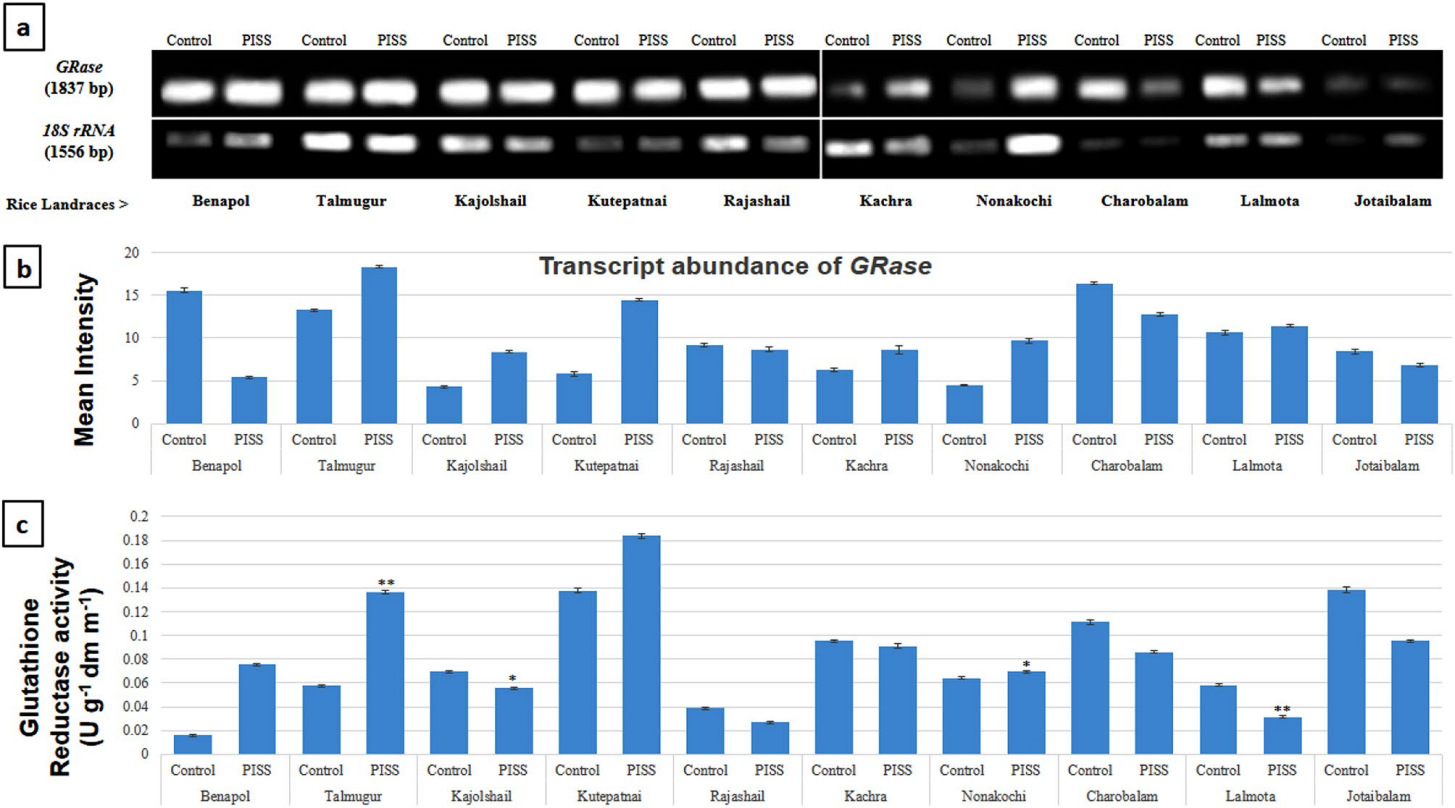
Comparative transcript abundance of gene *GRase*  from untreated control and PISS (200 mM NaCl) raised seedling of ten experimental land races of rice of Sundarban, Bangladesh (**a**). Semi – quantitative RT–PCR was performed as described in materials and methods. The rice 18S rRNA control reaction below each set of corresponding gene reactions were conducted on equivalent cDNA batches to verify equivalent loading reaction volumes on the gel. Antioxidant genes were amplified for 25 cycles. Borders in the gel images represent the areas in which they are juxtaposed from two different gels. (**b**) Bar diagram represents mean intensity of relative expression of genes. (**c**) Activity of the corresponding enzyme glutathione reductase of PISS (200 mM NaCl) raised seedling of ten experimental land races of rice.

**Figure 5:**
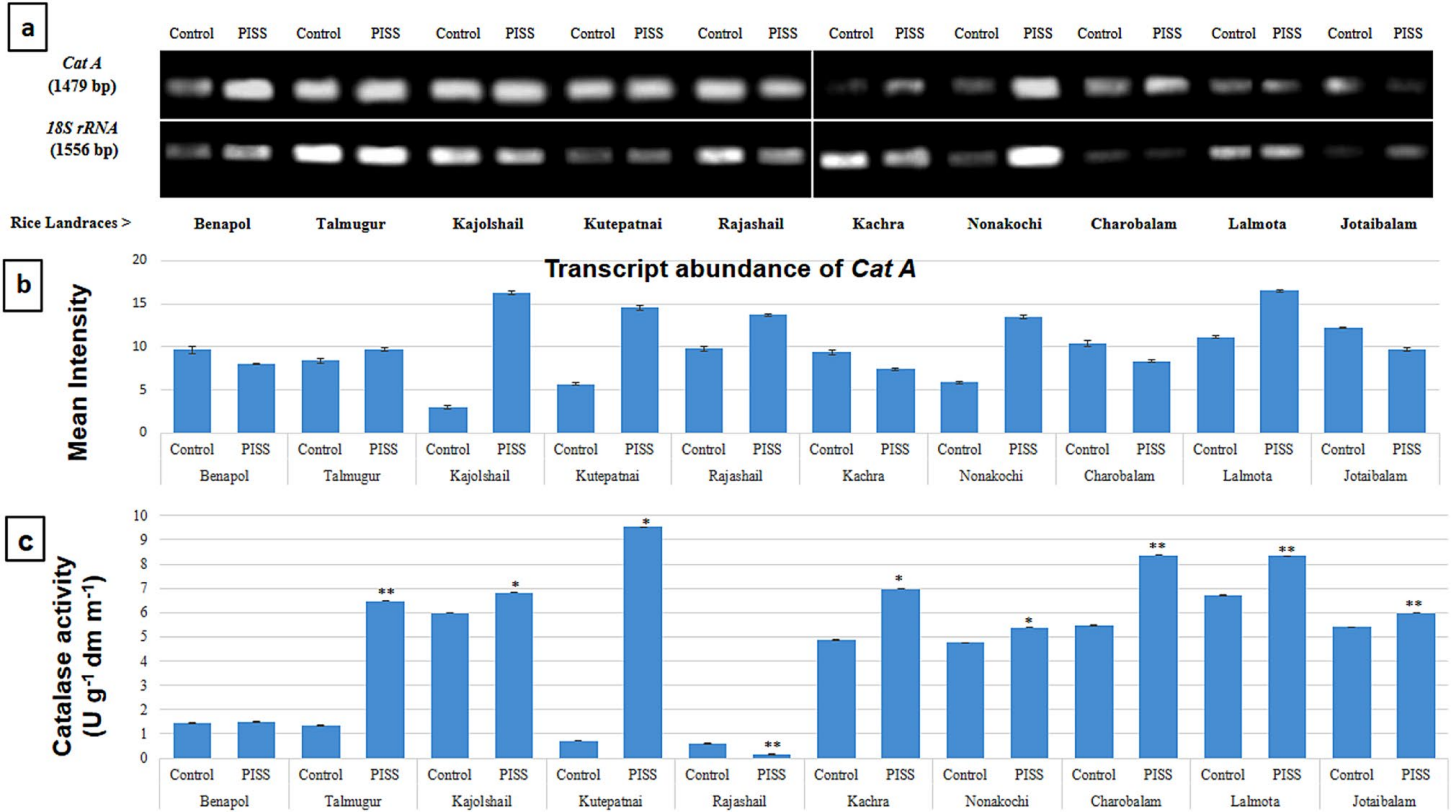
Comparative transcript abundance of gene *CatA *from untreated control and PISS (200 mM NaCl) raised seedling of ten experimental land races of rice of Sundarban, Bangladesh (**a**). Semi – quantitative RT–PCR was performed as described in materials and methods. The rice 18S rRNA control reaction below each set of corresponding gene reactions were conducted on equivalent cDNA batches to verify equivalent loading reaction volumes on the gel. Antioxidant genes were amplified for 25 cycles. Borders in the gel images represent the areas in which they are juxtaposed from two different gels. (**b**) Bar diagram represents mean intensity of relative expression of genes. (**c**) Activity of the corresponding enzyme catalase of PISS (200 mM NaCl) raised seedling of ten experimental land races of rice.

Semi – quantitative RT-PCR employed here provided the data of relative transcript abundance, therefore reflecting the level of gene expression under PISS *vis-à-vis* their untreated control seedlings of all experimental landraces (Figs. 1, 2, 3, 4 and 5). The transcripts encoding the genes of H_2_O_2_ forming enzyme (*RboH*) and processing enzymes involving A-G and CAT pathways [superoxide dismutase (SOD), ascorbate peroxidase (APOX), glutathione reductase (GR), catalase (CAT)] that effectively determine the turnover of ROS H_2_O_2_ were assessed relative to the *18S rRNA* using semi quantitative RT-PCR from post-imbibitional salinity stress (PISS) raised seedlings of all experimental landraces. *18S rRNA* transcript remained almost unchanged in both control and PISS raised seedlings of experimental rice and hence selected as suitable gel-loading buffer for transcript analysis of H_2_O_2_ turnover system (*RboH, SodCc2, OsAPx2, GRase, CatA*) coding respectively for apoplastic NADPH-oxidase, cytosolic Cu/Zn SOD, APOX, GR and CAT.

Imposition of PISS to the magnitude of 200 mM NaCl (EC-0.18 Scm^−1^) caused differential transcriptional regulation of the gene of the enzyme RboH (NADPH-oxidase) for the 10 selected experimental landraces of rice (Fig. 1). There was a significant up-regulation of the expression of the gene *RboH* for the landraces Kajolshail, Talmugur, Kutepatnai, Rajashail, Benapol, Jotaibalam and Charobalam. The landrace Kachra though exhibited an up-regulation in their expression but unlike the others the increment is marginal. On the contrary the landraces like Nonakochi and Lalmota exhibited a down-regulation in the expression of *RboH*. Assessment of the activity of the corresponding enzyme RboH, however, revealed more or less the same trend except for the landrace Kutepatnai. Comparative assessment of the activity of the enzyme NADPH-oxidase revealed significant up-regulation for the landraces Talmugur, Jotaibalam, Benapol, Charobalam, Kutepatnai and Rajashail. The landraces Nonakochi, Lalmota and Kachra on the other hand showed a significant down-regulation in their activities, corroborating well with the transcriptome data. So, overall, the expression of the gene *RboH* and the activity of the same enzyme in general exhibited a salinity induced up-regulation for most of the landraces, except a few (Fig. 1).

Comparative assessments of transcript abundance of the gene *SodCc2* responsible for the synthesis of the enzyme SOD associated with dismutation of superoxide (O_2_^•-^) in general, exhibited a mixed response under PISS for all the experimental landraces of rice studied. The landraces like Benapol, Talmugur, Kutepatnai, Kajolshail, Rajashail and Jotaibalam exhibited transcriptional up-regulation showing greater transcript abundance of the gene for their corresponding salinity stress-raised seedlings (Fig. 2). On the contrary, the landraces like Kachra, Nonakochi, Charobalam and Lalmota exhibited down-regulation showing reduced transcript abundance of the same gene for their seedlings raised under salinity stress. When we compared the activity of the same enzyme SOD, it exhibited more or less same trend as that of their transcript level, with an up-regulation of their activities under NaCl salinity stress for the landraces Talmugur, Kutepatnai, Kajolshail, Benapol and Jotaibalam have been noticed. The landrace Kachra which showed a significant down-regulation in its transcript abundance of *SodCc2* gene also exhibited a significant reduction in the activity of the same enzyme whereas the landraces Nonakochi, Charobalam and Lalmota which exhibited a marginal reduction in transcript abundance of the gene *SodCc2* showed negligible changes in the activities of the corresponding enzyme hinting at different level of regulation of the enzyme activity (Fig. 2).

Assessment of transcript abundance of the gene of APOX (*OsAPx2*) of salinity stress-raised seedlings in general showed a mixed response. The landraces Talmugur, Kutepatnai, Rajashail, Jotaibalam and Benapol exhibited salinity induced transcriptional up-regulation of *OsAPx2* gene. Whereas the landraces like Kajolshail, Kachra, Nonakochi, Lalmota exhibited down-regulation of *OsAPx2* expression. However, the maximum extent of up-regulation of expression of *OsAPx2* was noticed for the landraces Talmugur, Rajashail and Jotaibalam under PISS (Fig. 3). Corresponding assessment of the activities of APOX for all ten experimental landraces of rice, more or less revealed the same trend as that of transcript abundance for the landraces Talmugur, Kutepatnai, Charobalam, Nonakochi, Lalmota and Kajolshail. Both the landraces Talmugur and Kutepatnai exhibited up-regulation in the activities of APOX corroborating well with the data of gene expression of *OsAPx2*. The landraces Benapol, Rajashail and Jotaibalam through exhibited significant transcriptional up-regulation of *OsAPx2* gene but when we assessed the activities of APOX enzyme, it showed different trend (down-regulation) hinting at the involvement of post-translational regulation of gene expression (Fig. 3).

The transcript abundance of the gene *GRase* of the seedlings of the landraces Talmugur, Kutepatnai, Kajolshail and Nonakochi showed a sharp rise whereas salinity stress-raised seedlings of Benapol, Rajashail, Charobalam and Jotaibalam showed a marked decline in transcript abundance of *GRase* genes. The landraces Rajashail, Kachra, Lalmota showed no appreciable changes in transcript abundance of *GRase* genes of salinity stress-raised seedlings as compared to their untreated control (Fig. 4). Comparative assessment of the activity of GR enzyme in seedlings raised from salinity stress treatment also revealed more or less the same pattern of changes as their transcript abundance of the genes raised under salinity stress. Both the cultivars Talmugur and Kutepatnai exhibited significant rise in activity of GR, corroborating well with the data of transcript abundance of their correspondent genes. The significantly reduced activities of Jotaibalam, Lalmota, and Charobalam also correspond with their gene expression data (Fig. 4).

The gene of the antioxidative enzyme CAT (*CatA*) also showed a strong up-regulation in their expression for the landraces Kutepatnai, Kajolshail and Nonakochi. The landraces Benapol, Kachra, Charobalam and Jotaibalam showed a clear down-regulation in transcript abundance of the gene *CatA* for the seedling rose under salinity stress (Fig. 5). Comparative assessments of activities of CAT also substantiate the data of the corresponding transcript abundance, showing a significant up-regulation for Kutepatnai, Talmugur and Charobalam. The activities of enzyme CAT, however, showed its down-regulation for the landraces Rajashail, whereas the cultivars Nonakochi, Jotaibalam, Kajolshail and Benapol showed only insignificant marginal increment (Fig. 5).

**Figure 6:**
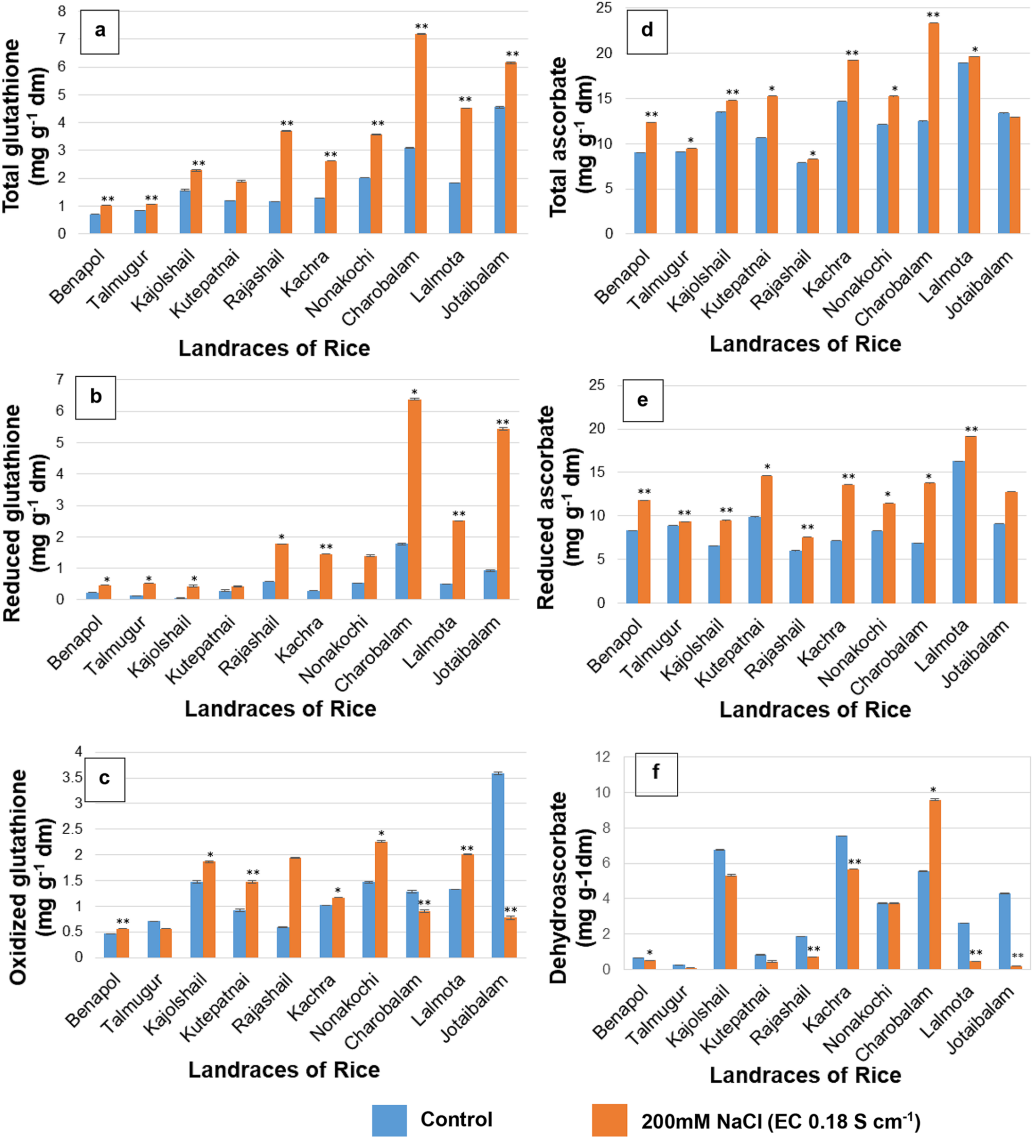
Comparative assessment of changes in redox turnover of Ascorbate and Glutathione pull (assessed in terms of total ascorbate, reduced ascorbate, dehydroascorbate and total glutathione, reduced glutathione and oxidized glutathione) of PISS (200 mM NaCl) raised seedling of ten experimental land races of rice of Sundarban, Bangladesh. Results are mean of three replicates ± standard error. ** represents significant changes from control at 0.01 level (t-test) and * represents significant changes from control at 0.05 level (t-test).

PISS in general up-regulates the accumulation of total glutathione pool for all ten rice landraces studied, though the extent of enhancement varies among the landraces (Fig. 6). However, the redox turnover dynamics assessed in terms of GSH/GSSG pool, revealed a significant rise for all the landraces except Nonakochi and when we compared the salinity induced changes of reduced (GSH) and oxidized (GSSG) pool of glutathione, we find the balance is tilted more towards reduced form as that of the oxidized form for most of the landraces of rice we have studied hinting at the greater turnover dynamics of the reduced form of glutathione under PISS. The significant increment of oxidized glutathione (GSSG) for the landraces like Lalmota, Nonakochi, Rajashail and Kachra, signify the reduced pace of the A-G pathway concomitant with the data of the transcript abundance and activities of the enzymes of A-G cycle. On the contrary, greater abundance of reduced form of glutathione (GSH) signifies the greater turnover of A-G pathway for the successful buffering of endogenous H_2_O_2_ formed under PISS (Fig. 6).

Comparative estimation of total, reduced (AsA) and oxidized (DHA) pool of ascorbate in the seedlings of experimental landraces of rice grown under PISS revealed a landrace-specific difference in their accumulation. There is in general significant increment of total pool of ascorbate for the landraces Kutepatnai, Benapol, Talmugur, Charobalam and Kachra under PISS. The landraces Rajashail, Jotaibalam on the other hand showed no significant changes in the accumulation of total pool of ascorbate. Estimation of the comparison of the ratio of reduced/ oxidized pool of ascorbate (AsA/DHA) though revealed a tilt more towards reduced form under PISS but when compared, the landraces Kutepatnai, Nonakochi, Kachra and Benapol exhibited more increment of the reduced form as that of their oxidized form. The landrace Kutepatnai and Talmugur in particular exhibited a reduced accumulation of DHA (oxidized pool of ascorbic acid), hinting at more turnover of the oxidized form towards the regeneration of reduced form through enhancement of pace of A-G cycle, corroborating well with the gene expression and enzyme activities data of Halliwell-Asada pathway (Fig. 6).

### Redox metabolic shift in PISS-raised seedling of ten rice landraces of coastal areas of Sundarban, Bangladesh

**Figure 7:**
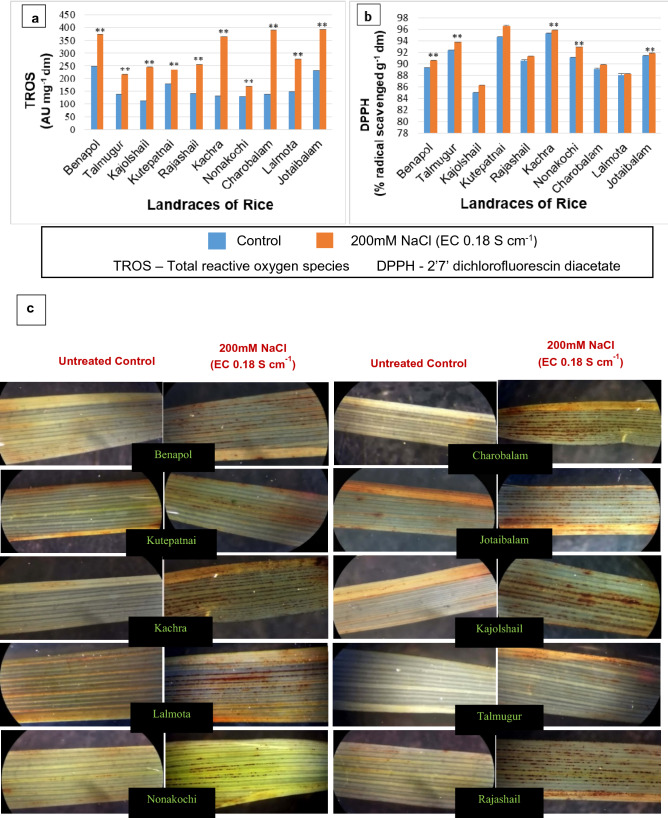
Assessment of PISS-induced changes in redox homeostasis [monitored in terms of total ROS accumulation (DCFDA oxidation) and total antioxidant capacity (DPPH radical scavenging property)] along with histochemical in situ localization of ROS (H_2_O_2_ ) through DAB staining in ten experimental land races of rice of Sundarban, Bangladesh. Results are mean of three replicates ± standard error. **Represents significant changes from control at 0.01 level (t-test) and * represents significant changes from control at 0.05 level (t-test).

**Figure 8:**
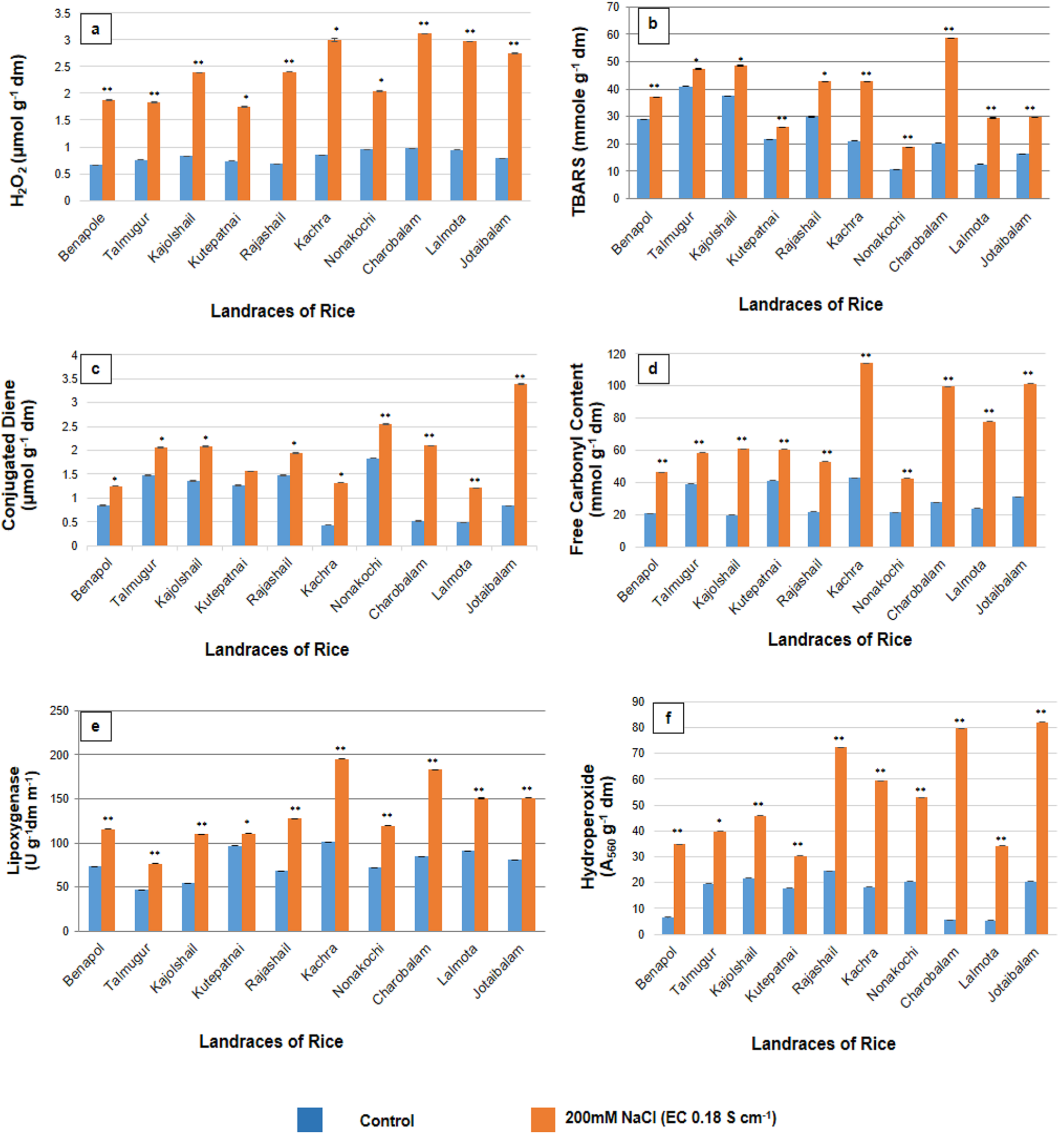
Assessment of PISS-induced changes in quality redox biomarkers assessed in terms of parameters of oxidative damage of membrane protein(free carbonyl content), lipid (accumulation of hydroperoxide, conjugated diene, TBARS and activities of lipoxygenase) in ten experimental land races of rice of Sundarban, Bangladesh. Results are mean of three replicates ± standard error. **Represents significant changes from control at 0.01 level (t-test) and *Represents significant changes from control at 0.05 level (t-test).

Assessment of pro-oxidants in terms of oxidation of DCFDA (Total ROS) and H_2_O_2_ along with the *in-situ* localization of ROS through DAB (3,3’-diaminobenzidine) staining for all the experimental landraces of rice raised under NaCl PISS revealed a clear shift of redox status towards pro-oxidants (Figs. 7, 8). Comparative assessment of accumulation of total ROS [through spectrofluorometric assessment of oxidation of DCFDA (2’, 7’-dichlorofluorescindiacetate)] and H_2_O_2_ revealed the landrace specific accumulation under PISS (Figs. 7, 8). The landraces Kutepatnai, Talmugur and Nonakochi though exhibited an up-regulation in the accumulation of total ROS and H_2_O_2_ but when compared with other landraces, their enhancement seems to be significantly lesser corroborating well with the data of enhanced efficacy of integrated RboH—A-G/CAT pathway and the transcriptional regulation of the genes involved in the said pathway. The landraces Kutepatnai, Nonakochi and Talmugur exhibited 30.522% 31.013% and 57.661% increment in the accumulation of total ROS vis-à-vis 184.439%, 180.668% and 70.628%, increment for Charobalam, Kachra and Jotaibalam respectively. In situ localization of H_2_O_2_ by DAB staining further substantiate the landrace specific accumulation in experimental landraces of rice, showing the same trend of result as that of the endogenous level of H_2_O_2_ and total ROS. The seedlings of the landraces Charobalam, Kachra, Jotaibalam, Kajolshail, Lalmota and Rajashail raised under PISS exhibited greater intensity of staining in the leaf blades, spreading almost throughout the major surfaces as compared to their untreated control. On the contrary, seedlings rose from PISS for the landraces Talmugur, Kutepatnai and Nonakochi in particular, the intensity of staining is not only faint but also found to be localized and patchy on the corresponding leaf blade suggesting histochemical evidences of differential accumulation of pro-oxidants for the experimental landraces of rice (Figs. 7, 8).

Further the cumulative antioxidant capacity assessed in terms of DPPH (2, 2^/^-diphenyl-1-pycryl hydrazyl) radical scavenging assay exhibited a general trend of increment for most of the seedlings of the experimental landraces of rice developed under PISS (Fig. 7). However, when compared, the landraces Kutepatnai, Talmugur and Nonakochi exhibited remarkably greater DPPH radical scavenging property as compared to others under the same magnitude of PISS suggesting greater role of antioxidative defense competence in regulating the endogenous titer of ROS under PISS. The landraces like Charobalam, Kachra, Jotaibalam, Lalmota and Rajashail exhibited only a marginal increment in DPPH radical scavenging property confirming incompetence in redox-regulation under PISS, supporting well with the data of the efficiency of A-G pathway and their corresponding gene expression.

### Oxidative deterioration products as sensitive redox biomarkers in PISS-raised seedling of ten rice landraces of coastal areas of Sundarban, Bangladesh

A comparative evaluation of enzymatic lipid peroxidation in general revealed an increment under PISS for all landraces of rice studied. But when we compared, the accumulation of the lipid peroxidation product in terms of conjugated diene (CD), hydroperoxide (HPOX) and thiobarbituric acid reactive substances (TBARS), we find the inter landrace-specific differences (Fig. 8). The landraces Kutepatnai and Talmugur in particular exhibited significantly lesser extent of accumulation of all the products of membrane lipid peroxidation as compared to the other landraces. The landraces Kutepatnai and Talmugur exhibited 20.901% and 15.530% increment of TBARS and 24.332% and 39.567% increment of CD under PISS over their untreated control, whereas, the landraces Charobalam and Kachra exhibited 188.236% and 103.607% increment of TBARS and 303.660% and 203.926% increment of CD under PISS in comparison to their untreated control. These data of the accumulation of membrane lipid peroxidation products strongly vouch the differential capacity of the landraces to combat salinity induced oxidative threat with the increasing order Kutepatnai, Talmugur and Nonakochi > Jotaibalam, Kachra and Charobalam (Fig. 8).

The same trend of result was observed when we compared the free carbonyl content (RC = O) of salinity induced post-imbibitional seedlings of experimental rice landraces raised under PISS showing significantly lesser extent of accumulation of the same for the landraces Kutepatnai, Talmugur and Nonakochi as compared to other landraces, corroborating once again the data of endogenous redox cue, shift in redox metabolism and transcript abundance of the genes of the enzyme of central hub of antioxidants (Figs. 1, 2, 3, 4, 5, 6, 7 and 8).

### Changes in ion homeostasis, germination and early growth phenotypes in PISS-raised seedling of ten rice landraces of coastal areas of Sundarban, Bangladesh

A comparative evaluation of changes in ion homeostasis (assessed in terms of the changes in Na^+^/K^+^ ratio and accumulation of Cl^-^ in PISS raised seedlings of experimental rice landraces revealed strong correlation between loss of redox homeostasis with ion accumulation and relative water content (Supplementary Table 3). The landraces Kutepatnai, Talmugur and Nonakochi exhibiting better redox-regulatory abilities exploring central redox hub showed reduced Na^+^/K^+^ ratio and accumulation of Cl^-^ in PISS raised seedlings of experimental rice landraces as compared to other land races.

A comparative cumulative analysis of all important germination phenotypes (as recommended by ISTA) revealed a differential response to PISS with the landrace Kutepatnai, exhibiting better germination and early growth phenotypes followed by Nonakochi and Talmugur under the identical salinity stress (see Supplementary Figs. 1 and 2 online). The other landraces like Benapol, Charobalam, Kachra, Rajashail, Jotaibalam and Lalmota exhibited a significant inhibition of germination as being noticed from all the parameters of germination phenotypes studied. The early growth performance assessed in terms of relative growth index (RGI) and vigor index (VI) also reflect the same results of germination phenotypes hinting at the role of delay or inhibition in seedling establishment for the landraces Benapol, Charobalam, Kachra, Kajolshail, Rajashail, Jotaibalam and Lalmota. The landraces Kutepatnai and Talmugur in particular exhibited better seedling establishment potentiality under the same magnitude of PISS (see Supplementary Fig. 1 and 2 online). Taking as a whole, the comprehensive germination phenotypes of PISS raised seedlings of ten experimental landraces of the coastal area of Sundarban, Bangladesh strongly support the role of internal redox cues as determinant of early growth performance. The PISS induced loss of redox homeostasis in the germinating tissue is found to be critical in determining the ability of the seed to germinate and establish successfully.

### Establishing relationships between germination phenotypes, quality redox biomarkers and salt tolerance classification using hierarchical cluster analysis

On the basis of germination and early growth performances and redox biomarkers, cluster analysis of ten rice landraces was studied and dendrogram was constructed based on the square Euclidean distance using “between group linkage” method as agglomerative clustering technique. The results obtained are presented in Supplementary Fig. 3. The positions of the ten landraces of rice in the dendrogram based on germination and early growth parameters were apparently distributed into three groups with Kutepatnai and Talmugur form first group and classified as salinity tolerant landraces. The land races Benapol, Nonakochi, Rajashail, Jotaibalam and Kajolshail form second group and Lalmota, Kachra and Charobalam form third group which can be classified as moderately tolerant and sensitive against salinity respectively. On the basis of coefficient value of agglomeration schedule, the highest similarity was observed between landraces Benapol and Nonakochi; Rajashail and Jotaibalam are also very much close to each other. Intense similarities were also observed between landraces Lalmota and Kachra, and again between Kutepatnai and Talmugur (see Supplementary Fig. 3a online).

The dendrogram based on the redox biomarkers in the stress induced conditions (PISS) grouped the rice landraces into two major groups with additional subgroups in each group presented in the Supplementary Fig. 3 online. Group one composed of seven rice landraces (Kutepatnai, Talmugur, Benapol, Kajolshail, Nonakochi, Rajashail and Jotaibalam) which had better performances against salinity. Group two consist of three landraces (Kachra, Charobalam and Lalmota) which showed sensitivity to salinity stress. Within the first group, Kutepatnai and Benapol are very much close to each other whereas in the second group, Kachra and Charobalam are also close to each other (see Supplementary Fig. 3b online).

## Discussion

The rice landraces of the coastal areas of Sundarban, Bangladesh, Kutepatnai and Talmugur though suffered PISS induced redox shift in metabolism, but when compared with the other experimental landraces raised under same magnitude of PISS, the redox shift seems to be significantly lesser. The redox tuning ability of these two landraces of rice in terms of regulation of redox status (pro-oxidant/antioxidant level) thus seems to be a determining factor for restricting the oxidative threat through protein oxidation and lipid peroxidation^5,25,26^. This differential outcome of redox status during early germination under PISS which eventually regulates differential oxidative threat and ion homeostasis to the experimental rice landraces concurs with the findings of Hossain and Dietz^5^, Stepien and Klobus^27^ and Liu et al.^28^. The differential oxidative threat suffered by experimental landraces of rice under PISS, assessed in terms of accumulation of CD, HPOX, TBARS, RC = O and lipoxygenase (LOX) activity, found to have a strong correlation with different germination phenotypes and seedling establishment (see Supplementary Tables 1, 2 and 4 online). Here we have used inclusive oxidative damage related parameters of membrane protein (RC = O) and lipid (CD, HPOX, TBARS and LOX) to corroborate the impact of PISS induced changes in redox status and ion homeostasis on germination and seedling establishment. Carbonylation of protein is always a sensitive redox marker or even a better oxidative stress index than peroxidation of lipid as the products of the later catabolize fast than that of the former^8,29^. The present study also implicates the significance of restoration of juvenile membrane system during the progress of germination by comparing different germination and early growth parameters under PISS induced oxidative threat. Usages of these inclusive parameters (germination rate, coefficient of velocity of germination, germination energy, germination rate index, mean germination time, relative growth index, T_50_ value, relative germination performance and vigor index) for the assessment of germination and early growth phenotypes of the experimental landraces not only explains PISS impact on germination impairment but also elucidates the role of oxidative threat on the progress of germination^21,30^. Though in the present study, the parameters of oxidative membrane damage (HPOX, CD, TBARS, RC = O and LOX activity) and early growth performances are not always completely linked or significantly correlated (see Supplementary Table 4 online) but in all cases it was noticed that the germination and early growth attributes is reciprocally regulated with greater accumulation of these components, hinting at the fact that the ability of PISS-tolerance needs significant mitigation of oxidative membrane protein and lipid damages^21,31–35^.

The present work explored integrated RboH-A-G cycle as the major determinant of redox hub under PISS, since the steady state level of H_2_O_2_ depends on the generation through RboH and its subsequent processing or buffering through A-G cycle^5,36,37^. So, any change in redox status of PISS-induced tissue is due to the relative efficacy of ROS generator and antioxidative defense systems through ROS regulatory network^5,8,36^. Several works on salinity induced ROS generating system suggests the role of NADPH-oxidase as primary pro-oxidant producer^5,38^. In fact, salinity stress up-regulates the membrane bound RboH depending on the magnitude of the stress imposed^39,40^. There is also report of salinity induced inhibition of RboH^41^. In our present study the landraces Lalmota, Nonakochi and Kachra exhibited down-regulation whereas the other landraces exhibited up-regulation in the activities of NADPH-oxidase under the same magnitude of NaCl salinity stress. The gene expression study of the same enzyme (RboH) also revealed almost the same pattern with Lalmota and Nonakochi showed lesser transcript abundance under PISS. The significant transcriptional up-regulation of the gene *RboH* for the landraces Talmugur, Kajolshail and Kutepatnai corresponds with their enzymatic activity under PISS. Other landraces like Benapol, Rajashail, Charobalam and Jotaibalam exhibited only marginal enhancement of the activity of NADPH-oxidase along with their corresponding transcriptional up-regulation of the concerned gene. The differential expression of the gene *RboH* and the activity of the corresponding enzyme under the same magnitude of salinity stress in the experimental rice landraces may be explored by the plant differentially with their subsequent buffering through redox scavenging system for regulation of salinity stress^5,36,40^.

Integrated SOD-A-G/CAT pathway being the most significant antioxidant hub for efficient redox buffering for buffering the endogenous titer of H_2_O_2,_ particularly under salinity stress^5,42,43^, we have explored this metabolism along with the transcriptional regulation of the genes involved not only to adjudge the redox regulation properties of the experimental landraces of rice but also utilize this as quality marker for screening the experimental landraces based on their salinity stress responsiveness. Over all, the reduced expression of both the genes *OsAPx2* and *GRase* or any one of the two makes the SOD-ascorbate–glutathione (SOD-A-G) cycle less efficient for scavenging H_2_O_2_ necessary for redox buffering and in this category we have Benapol, Kajolshail, Charobalam, Jotaibalam, Kachra, Nonakochi, Lalmota and Rajashail. On the contrary, Talmugur and Kutepatnai showed transcriptional up-regulation for both the genes *OsAPx2*, *GRase* under PISS, making them genetically competent to response to salinity stress for redox tuning necessary for ROS homeostasis^12,21,44^. Further, the transcript abundance of the other antioxidative defense gene *CatA* (coding CAT), on the other hand not only showed their enhanced level for the A-G pathway efficient landraces Kutepatnai and Talmugur but also showed salinity induced transcriptional up-regulation for other landraces like Kajolshail, Rajashail, Nonakochi and Lalmota, hinting at their better detox scavenging capacity of ROS through CAT.

Therefore, salinity induced transcriptional up-regulation of two important genes of A-G pathway (*OsAPx2* and *GRase*) along with *CatA* synergize the redox tuning ability of two landraces Kutepatnai and Talmugur causing efficient processing of H_2_O_2_ for the protection of cellular components from oxidative threat^5,44–46^. In fact, the analysis of integrated RboH-A-G/CAT pathway responsible for regulating turnover dynamics of H_2_O_2_ and fine tuning the redox-regulatory mechanism revealed significance of integrated dynamics of ROS generating and scavenging system rather than the efficiency of individual one. So, considering this as an important contrivance of redox regulation, the landraces Kutepatnai and Talmugur stands in better position to combat salinity stress during early germination^34,44,47^. RboH-A-G/CAT pathway thus corresponds to one of the key redox tuning mechanisms in plants under salinity which is primarily responsible for processing of the prominent ROS H_2_O_2_, thereby, restore redox-homeostasis and bypass the origin of lethal oxidative stress, as evident from the data of oxidative protein and lipid damages^5,12,44,47^.

The comparative study of redox couple (GSH/GSSG and AsA/DHA) exhibited better redox turnover dynamics of glutathione and ascorbate for maintenance towards reduced state for the landraces Kutepatnai and Talmugur, further substantiating the data of better redox tuning ability necessary associated with ROS homeostasis under salinity and the result of hierarchical cluster analysis for the determination of salinity tolerance^5,12,22,44,46^.

Taken as a whole, the gene expression data and the metabolic fingerprints of central redox hub (RboH-A-G/CAT pathway), pro-oxidant/antioxidant status, parameters of oxidative damages of membrane protein and lipid) and the corresponding germination phenotypes strongly vouch the significance of redox tuning at metabolic interface for combating salinity stress. Further, these comprehensive parameters of central redox hub and other oxidative deterioration products and germination phenotypes may be explored as standard physiological and molecular redox fingerprints for screening salinity resistant rice landraces.

## Materials and methods

### Plant growth and treatment of NaCl to induce post-imbibitional salinity stress

Though several land races of rice are generally cultivated in coastal regions of Sundarban Bangladesh, a wide variation of physiological response towards salinity stress has been observed, necessitating quality trait-based screening of salt tolerant rice landraces for crop improvement. In this backdrop, seeds of ten landraces of rice (*Oryza sativa* L., landraces Nonakochi, Kajolshail, Rajashail, Benapol, Kachra, Lalmota, Talmugur, Kutepatnai, Charobalam and Jotaibalam), selected as experimental material, have been collected from BARSIC (Bangladesh Resource Center for Indigenous Knowledge), Munsiganj, Satkhira, Bangladesh. Seeds of the rice were washed with distilled water and were treated with 0.2% HgCl_2_ for 5 min and then washed thrice with sterile distilled water. The surface sterilized seeds were imbibed in distilled water for 48 h in darkness at 25° ± 2 °C and thereafter, were sown on moist filter paper in petri plates and were placed in standardized conditions of thermostat-controlled seed germinator cum stability chamber (Remi 82 BL, India) maintained at 25° ± 2 °C. For imposing post-imbibitional salinity stress (PISS), one of the two different water imbibed seed lots was treated with 200 mM NaCl for 7 days (magnitude of the NaCl salinity was selected based on dose–response results of pilot experiment), with intermittent changes of treating solutions in petri plates (24 h interval). For standardizing PISS treatment conditions, water imbibed seed lots were treated with different magnitude of NaCl concentrations (50 mM, 100 mM, 150 mM, 200 mM and 250 mM). After the imposition of PISS, germinating seeds were allowed to grow for next 168 h in Plant Growth Chamber (LAB-X, India), maintained at temperature 25 °C ± 2 °C, relative humidity 65 ± 2% and 14-h photoperiod with 270 µm m^-2^ s^-1^ illumination. Subsequently, quality redox parameters [accumulation of pro-oxidant (H_2_O_2_), radical scavenging property (DPPH assay) and oxidative protein damage (free carbonyl content)] and standard germination and early growth phenotypes (T_50_ value and relative growth index) were estimated for evaluating the overall optimum impact of different magnitude of PISS. Considering results of all the parameters and overall optimum significant responses, we have standardized 200 mM NaCl salinity stress as treatment concentration for imposing PISS [Supplementary Tables 1 and 2 online]. Subsequently all experiments were done with 200 mM NaCl PISS. All the seed lots were allowed to grow at 25°  ± 2 °C with 14 h photo period (light intensity 270 µmol m^−2^ s^−1^) and 78 ± 2% relative humidity after imposing PISS. For all biochemical analysis, 168 h old PISS-raised seedlings raised from aforesaid conditions were used.

### Determination of reactive oxygen species (ROS) and total antioxidant capacity (DPPH radical scavenging property)

#### Estimation of Total ROS

For estimation of total ROS, protocol of Simontacchi et al.^48^ was followed. 30 mg of seedling tissue was incubated for 60 min at 30 °C in 100 µM solution of 2’, 7’-dichlorofluorescindiacetate (DCFDA, Sigma) dissolved in TRIS–HCl (p^H^-7.0). Spectrofluorometric determination of the fluorescence in the supernatant was then monitored with excitation at 488 nm and emission at 525 nm. For the control set, same process was followed without DCFDA to differentiate ROS from other long-lived substances able to react with DCFDA. After 60 min, tissue was removed and DCFDA was added in TRIS–HCl for further 60 min incubation before monitoring fluorescence. Final fluorescence value of ROS was measured through subtraction between the two values. Another parallel blank (assay mixture without tissue) was prepared and monitored fluorescence as correction factor for auto-fluorescence.

#### In situ localization of H_2_O_2_ through DAB staining

For the DAB assay, the 7 days old leaf segments of germinating seedlings were dipped into 2 ml of DAB (3, 3’diaminobenzidine) stain (1 mg/mL prepared in double-distilled water, pH 3.8) in a watch glass and incubated for 8 h at room temperature under light. After incubation, the leaf segments were dipped in the absolute ethanol and then kept in a water bath (100°C) until the tissue became colourless. Then the samples were mounted on slides with 20% glycerol. The image was captured using a stereo microscope (Leica Wild M3B stereo zoom microscope, Germany) and the presence of H_2_O_2_ was spotted as brown or deep brown dotted spots in the leaf tissue.

#### Estimation of H_2_O_2_ generation

Hydrogen peroxide was estimated by the procedure of MacNevin and Uron^49^ using titanic sulfate. For this, 1 g of tissue was extracted with 5 mL of cold acetone and filtered through Whatman No. 1 filter paper and volume made up to 10 mL with distilled water. Then 1 mL of 5% titanic sulfate (in 20% H_2_SO_4_) was added to this, which was followed by addition of 2 mL of concentrated NH_4_OH and finally centrifuged at 6000 rpm for 10 min. Pellet obtained was washed with 5 mL of acetone (thrice) and then centrifuged at 5000 rpm for 10 min. Then, the pellet was dissolved in 3 mL of 2(N) H_2_SO_4_ and absorbance was taken at 420 nm against a blank.

#### DPPH (2, 2/-diphenyl-1-pycryl hydrazyl) radical scavenging activity

DPPH radical scavenging property was determined by following the procedure of Mensor et al.^50^. The dry seedling tissue were extracted through incubation in 80% methanol at 28 °C for 24 h in shaking incubator. After centrifugation at 3500 rpm for 20 min in cold, collected supernatant was filtered and used for DPPH radical scavenging activity mixed with DPPH solution (0.04 mg mL^−1^ ethanol) in 1:3 ratios. Absorbance at 517 nm was observed after 30 min incubation in dark with the help of UV–VIS spectrophotometer (Shimadzu, Model UV-1900i). Finally total antioxidant capacity (TAC) was determined by the following formula:

$${\text{TAC }}\left( \% \right) \, = \left[ {1 - \frac{{{\text{Ai}} - {\text{Aj}}}}{{{\text{Ac}}}}} \right] \, \times {1}00$$

$$\begin{gathered} [{\text{A}}_{{\text{i}}} = \, 1\;{\text{mL}}\;{\text{sample}}\;{ + }\;3\;{\text{mL}}\;{\text{DPPH}}; \hfill \\ {\text{A}}_{{\text{j}}} = \, 1\;{\text{mL}}\;{\text{sample}}\;{ + }\;3\;{\text{mL}}\;{\text{ethanol}};{\text{A}}_{{\text{c}}} = \, 1\;{\text{mL}}\;{\text{ethanol}}\;{ + }\;3\;{\text{mL}}\;{\text{DPPH}}] \hfill \\ \end{gathered}$$

### Determination of indices of oxidative membrane damage (estimation of hydroperoxide, conjugated diene, thiobarbituric acid reactive substances, activity of lipoxygenase and estimation of free carbonyl content)

#### Hydroperoxide

To estimate Hydroperoxide, 500 mg of tissue was homogenized in 150 mM Tris HCl (P^H^-6.8), centrifuged at 5000 rpm and subsequently assayed. The reaction mixture contained 250 mM ammonium ferrous sulfate, 100 mM xylenol orange, 0.25 mM H_2_SO_4_, 4 mM BHT prepared in 90% (v/v) methanol and an aliquote of the sample. After 30 min of incubation at room temperature, the reaction mixture was added with 100 mM triphenyl phosphine to specifically reduce hydroperoxide to distinguish from H_2_O_2_^51^. Absorbance was taken at 560 nm with the help of UV–VIS spectrophotometer (Shimadzu, Model UV-1900i).

#### Conjugated diene

For estimating conjugated diene, the process of Buege and Aust^52^ was followed. 500 mg of tissue, extracted with chloroform: methanol mix (2:1) was followed by vigorous vortexing and centrifugation at 2000 rpm for10 min. The upper layer obtained was discarded along with the proteins, while the lower chloroform layer was dried under a stream of nitrogen at 45 °C. The residue obtained was dissolved in cyclohexane and absorbance was taken with the help of UV–VIS spectrophotometer (Shimadzu, Model UV-1900i) at 230 nm against a cyclohexane (standard 1 O. D. = 37.5 n moles).

#### Thiobarbituric acid reactive substances

To estimate membrane lipid peroxidation, test for thiobarbituric acid reactive substances (TBARS) was performed using the procedure of Heath and Packer^53^. 200 mg of sample was homogenized in 5 mL 0.1% trichloroacetic acid (TCA) and then centrifuged at 10,000 rpm for 15 min and finally supernatant was taken. To 1 mL of supernatant, 3 mL of 5% TCA containing 1% thiobarbituric acid (TBA) was added and heated in a hot water bath for 30 min and cooled quickly in cold water bath. It was finally centrifuged at 10,000 rpm for 10 min. The absorbance of the supernatant was measured at 530 nm with the help of UV–VIS spectrophotometer (Shimadzu, Model UV-1900i). The concentration of TBARS was measured from its extinction coefficient of 155 µM cm^-1^. The non-specific turbidity was corrected by subtracting A_600_ from A_530_ value. The formula employed as:

$${\text{Conc}}{.}\;{\text{of}}\;{\text{unknown}} = \frac{{{\text{Absorbance }}\;{\text{of}}\;{\text{unknown}}\;{\text{at }}\;{530}\;{\text{nm }}\;{\text{mols/l}}}}{{{\text{Diameter}}\;{\text{of }}\;{\text{cuvette }} \times {155}}}$$

The TBARS content was finally expressed in n mol g^-1^ dry mass of tissue.

#### Lipoxygenase activity

Lipoxygenase activity was determined according to Peterman and Siedow^54^. 200 mg of tissue was homogenized with 5 mL of 50 mM Na-phosphate buffer (p^H^-6.5) and centrifuged at 5000 rpm for 5 min. Supernatant was taken and re-centrifuged at 17,000 rpm for 10 min in cold. The reaction mixture contained 1 mL of 1.65 mM Na-phosphate buffer (P^H^-6.5) and 1 mL of 1.3 mM linoleic acid. After 1 h of incubation at 25 °C, absorbance was read at 234 nm with the help of UV–VIS spectrophotometer (Shimadzu, Model UV-1900i).

#### Free carbonyl content

Oxidative damage to proteins was estimated as the content of carbonyl groups following the procedure of Jiang and Zhang^55^. 500 mg of tissues (root and shoot) were homogenized with 3 mL of 50 mM potassium phosphate buffer (pH 7.0) containing 1 mM EDTA (ethylenediaminetetraacetic acid), 1 mM PMSF (phenylmethylsulfonyl fluoride), 10 mM DTT (dithiothreitol) and 5 µg mL^-1^ leupeptin (protease inhibitors). The homogenate was centrifuged at 15,000 × g for 25 min and the supernatant was made free from contaminating nucleic acids by treatment with streptomycin sulfate. An equal volume of 10 mM DNPH in 2 M HCl was added to supernatant containing the oxidized protein. These were allowed to stand in the dark at room temperature for 1 h, with vortex in every 10 min intervals. Samples were precipitated with trichloroacetic acid (TCA; 20% final concentration) and centrifuged in a table-top micro centrifuge for 5 min. The supernatants were discarded and the protein pellets were washed twice more with TCA and then washed three times with 1 mL portions of ethanol/ethylacetate (1:1) to remove any free DNPH. The protein samples were re-suspended in 1 mL of 6 M guanidine hydrochloride (dissolved in 20 mM phosphate buffer, pH 2.3) at 37 °C for 15 min with vortex mixing. Carbonyl contents were determined from the absorbance at 370 nm by spectrophotometer (Shimadzu, Model UV-1900i)using a molar absorption coefficient of 22 mM cm^−1^.

### Extraction and estimation of enzymatic antioxidants

1 g of tissue was homogenized with 5 mL 0.1 mM potassium phosphate buffer (p^H^-7.0) containing 5 mM Na-acetate and the homogenate was centrifuged at 20,000 g for 30 min at 4 °C. 20 µL of enzyme extract was mixed with a total reaction mixture of 500 µL containing 100 mM Na-acetate (p^H^-6.5), 1 mM MnCl_2_ and 0.5 mM paracoumaric acid containing 0.2 mM NADPH. Oxidation of NADPH was measured by the decrease in absorbance at 340 nm with the help of UV–VIS spectrophotometer (Shimadzu, Model UV-1900i).

The extraction and estimation of superoxide dismutase (SOD) activity was determined by measuring the photochemical reduction ability of nitroblue tetrazolium (NBT) according to Giannopolities and Ries^56^ with some necessary modifications. The reaction mixture contained 0.05 M Na_2_CO_3_, 0.1 mM EDTA, 10 mM NBT and 13 mM riboflavin. The assay mixture was placed under 40 W florescent lamps at a distance of 30 cm at 25 °C for 30 min and absorbance was read at 560 nm. The non-irradiated sample served as control.

Ascorbate peroxidase (APOX) activity was determined according to Nakano and Asada^57^ using homogenates previously supplemented with 0.5 mM ascorbic acid and 0.1 mM EDTA. Parallel experiments in presence of p-chloromercuribenzoate (50 µM) were performed to rule out any interference from guaiacol peroxidases.

Glutathione reductase (GR) activity was measured according to Schaedle and Bassham^58^. The reaction mixture contained 50 mM Tris–HCl (pH 7.6), 0.15 mM NADPH, 0.5 mM oxidized glutathione (GSSG), 3 mM MgCl_2_ and 100 µL homogenate (7 mg protein mL^-1^). NADPH oxidation was measured at 340 nm. The enzyme activity in all cases was expressed as enzyme unit min^-1^g^1^dm according to Fick and Qualset^59^.

For the extraction and estimation of catalase (CAT), the process of Snell and Snell^60^ was followed with some necessary modifications. 100 mg of tissue was homogenized with 0.1 M sodium phosphate buffer (pH 7.0) containing 1% polyvinylpolypyrillidone (PVPP) and the homogenate was centrifuged at 5000 rpm for 10 min at 4 °C. For enzyme assay, 1 mL of enzyme extract was added to 1 mL of 0.5 mM H_2_O_2_ and incubated for 15 min at 37 °C. The reaction of the mixture was stopped by adding 2 mL 1% TiSO4 (in 25% H_2_SO_4_). The assay mixture was further centrifuged at 5000 rpm and the absorbance of the supernatant was read at 420 nm.

### Determination of components of ascorbate–glutathione pathway

1 g seedling tissue was homogenized in 10 mL cold 5% metaphosphoric acid. After centrifugation at 15,000 g for 30 min at 4 °C, the supernatant was collected for analyses of ascorbate and glutathione. This extraction procedure was slightly modified from the method given by Gossett et al.^61^. The measurement of total ascorbate and reduced ascorbate (AsA) contents was modified from the method of Law et al.^62^. Total ascorbate contents were determined in a 3 mL mixture. Enzyme extract was mixed with 10 mM DTT and 150 mM phosphate buffer (pH 7.4) containing 5 mM EDTA and was incubated at 25 °C for 10 min, followed by addition of 0.5% of N-ethylmaleimide. Then 10% TCA, 44% orthophosphoric acid, and 4% of α, α΄- bipyridyl were added. Finally, 3% of FeCl_3_ was added and the mixture was incubated in 40 °C for 40 min and the absorbance was detected at A_525_. AsA content was determined by adding distilled water instead of DTT and N-ethylmaleimide and then followed the same method as above. Total and reduced ascorbate contents were estimated from the standard curve of 0–100 µg mL^-1^ L-ascorbate determined by the above method. Oxidized ascorbate (DHA) content was calculated by the subtraction of AsA from total ascorbate.

Total glutathione content was determined by the absorbance at 412 nm according to the method, reported by Zhang and Kirkham^63^. The content of glutathione (reduced form) was estimated from the standard curve of 0–30 µmol mL^-1^ glutathione. After the removal of glutathione (GSH) by 2-vinylpyridine derivative, glutathione disulfide (GSSG) content was determined and the glutathione (GSH) content was calculated by the subtraction of glutathione disulfide (GSSH) content from total glutathione content.

### Estimation Na^+^, K^+^, and Cl^−^

Sodium, potassium and chloride ions were estimated by the process given in USDA hand book^64^ using flame photometric method. Dry samples were homogenized in 65% HNO_3_ and boiled for 30 min. After cooling 70% HClO_4_ was added and further boiled till white fumes vanished. Finally filtered and filtrate was taken and volume made upto 50 ml with distilled Milli Q water and reading was taken in Flame photometer and Ion meter respectively (Systronics, India).

### Determination of germination and early growth performances

For studying germination and early growth phenotypes, germination rate (GR), coefficient of velocity of germination (CVG), germination energy (GE), germination rate index (GRI), mean germination time (MGT), relative growth index (RGI), T_50_ value, relative germination performance (RGP) and vigor index (VI) were calculated as per the recommendation of ISTA (International Seed Testing Association) following the procedures of Rubio-Casal et al.^31^ and Bhattacharjee^42^.

Germination rate (GR) was calculated as:

$$\begin{aligned} {\text{GR}} = \, & {\text{number}}\;{\text{of}}\;{\text{germinated}}\;{\text{seed}}/{\text{days}}\;{\text{to}}\;{\text{first}}\;{\text{count }} + ......... + \\ & {\text{ number}}\;{\text{of}}\;{\text{germinated}}\;{\text{seeds/days}}\;{\text{to}}\;{\text{final}}\;{\text{count}}. \\ \end{aligned}$$

Mean germination time (MGT) was calculated as:

$$\begin{aligned} {\text{MGT}}\left( {\text{d}} \right) = & \Sigma {\text{NiTi}}/\Sigma {\text{Ni}};{\text{ where N is the number of seeds germinated on day T and}} \\ \, & {\text{T is time from the beginning of the germination test in terms of day}}. \\ \end{aligned}$$

Coefficient of velocity of germination (CVG) was calculated as:

$$\begin{aligned} {\text{CVG}} = \, & \Sigma {\text{Ni}}/\Sigma \left( {{\text{NiTi}}} \right) \times 100;\;{\text{where}}\;{\text{N}}\;{\text{is}}\;{\text{the}}\;{\text{number}}\;{\text{of}}\;{\text{seeds}}\;{\text{germinated}}\;{\text{every}}\;{\text{day}}\;{\text{and }} \\ & {\text{T}}\;{\text{is}}\;{\text{the}}\;{\text{number}}\;{\text{of}}\;{\text{days}}\;{\text{from}}\;{\text{seeding}}\;{\text{corresponding}}\;{\text{to}}\;{\text{N}}. \\ \end{aligned}$$

Germination rate index (GRI) was calculated as:

$$\begin{aligned} {\text{GRI}}\;{ = }\; & {\text{N}}1/1 + {\text{N}}2/2 + .... + {\text{Ni}}/{\text{i}};\;{\text{where}}\;{\text{N}}1\;{\text{is}}\;{\text{the}}\;{\text{germination}}\;{\text{percentage}}\;{\text{on}}\;{\text{day}}\;1, \, \\ & {\text{N2}}\;{\text{is}}\;{\text{the}}\;{\text{germination}}\;{\text{perentage}}\;{\text{at}}\;{\text{day}}\;{2;}\;{\text{and}}\;{\text{so}}\;{\text{on}}. \\ \end{aligned}$$

Germination energy (GE) was calculated as:

$${\text{GE}}\;{ = }\;{\text{No}}.\;{\text{of}}\;{\text{germinated}}\;{\text{seeds}}\;{\text{on}}\;4^{{{\text{th}}}} {\text{day}}/{\text{Total}}\;{\text{germinated}}\;{\text{seeds}}\;{\text{on}}\;{\text{final}}\;{\text{day}} \times 100$$

Relative growth index was calculated as:

$${\text{RGI}} = \left( {{\text{average}}\;{\text{dry}}\;{\text{mass}}\;{\text{of}}\;{\text{ten}}\;{\text{treated}}\;{\text{seedlings}}/{\text{average}}\;{\text{dry}}\;{\text{mass}}\;{\text{of}}\;{\text{ten}}\;{\text{control}}\;{\text{seedlings}}} \right) \times 100$$

Vigor index (VI) was measured as:

$${\text{VI}}\;{ = }\;\left( {{\text{Mean}}\;{\text{shoot}}\;{\text{length}}\;{ + }\;{\text{mean}}\;{\text{root}}\;{\text{length}}} \right)/{\text{Percentage}}\;{\text{of}}\;{\text{final}}\;{\text{germination}}.$$

Relative germination performance (RGP) was measured as:

RGP = (Percentage of germination under treatment /Percentage of germination under control) × 100

$${\text{T}}_{50} {\text{value}}\;{ = }\;{\text{Time}}\;\left( {\text{in hour}} \right)\;{\text{of}}\;50\% \;{\text{germination}}\;{\text{of}}\;{\text{seeds}}\;{\text{sown}}.$$

### RNA extraction and quantitative real-time polymerase chain reaction

Total RNA was extracted from cultured seedling of rice using the RNA-Xpress™ reagent (Himedia) based on the manufacturer’s instructions. The concentration and purity of RNA was determind by using an UV–Vis spectrophotometer. The first strand cDNA was synthesized using reverse transcriptase system (SuperScript™ III Reverse Transcriptase, Invitrogen) according to the manufacture’s protocol. Semi-quantitative RT-PCR was performed as described by Burch-Smith et al.^65^. For PCR, each reaction contained 2.0 µl of cDNA, 1 µl RT mix, 2.5 µl Taq buffer, 2 µl sense primer, 2 µl antisense primer, 1 µl dNTP mix, 1 µl Taq DNA polymerase, 2 µl MgCl_2_ (25 mM) and 13.5 µl diethyl pyrocarbonate (DEPC) treated deionized water. PCR conditions were as follows: initial denaturation of 2 min at 94 °C, followed by 30 cycles of denaturation for 1 min at 94 °C, annealing at 62 °C for 1 min, extension at 72 °C for 2 min, and a final extension of 10 min at 72 °C. 18S rRNA gene was used as a reference gene. Images of the RT-PCR products in ethidium bromide-stained agarose gels were acquired in BIO-Rad Molecular Imager Gel Doc XR system with high resolution CCD camera and quantification of bands was performed by Bio Rad image Densitometer GS-700 using quantity one software (version-4.6.9).

### DNA sequence of PCR primers were used for QRT-PCR determination of ascorbate–glutathione biosynthesis-related genes

**Table:** 

Name of the Genes	Accession no	Primer pairs	
*OsAPx2*	AK068430	F	5^/^-TCTTCCTGATGCCACACAAGGTTC -3^/^
		R	5^/^- TCCTTGTCACTCAAACCCATCTGC-3^/^
*GRase*	NM001055020	F	5^/^-CGACCTTTGACAGCACTGTTGG-3^/^
		R	5^/^-TGGTCAAGGTCCGCATTGTCAC-3^/^
*SodCc2*	L19434	F	5^/^-ATTGGCCGAGCTGTTGTTGTCC-3^/^
		R	5^/^-TAACCCTGGAGTCCGATGATT CCG-3^/^
*CatA*	EF371902	F	5^/^-AAGCTGTTCGTCCAGGTGATCG-3^/^
		R	5^/^-TGTCGACGTTGCGGTTGAGAAC-3^/^
*RboH*	J075145A22	F	5^/^-‍ACAGACCAGGGCAATTCAAC-3^/^
		R	5^/^-TCATCCACCCCTCTCACTTC-3^/^
*18S rRNA*	AB047313	F	5^/^- CTACGTCCCTGCCCTTTGTACA -3^/^
		R	5^/^- ACACTTCACCGGACCATTCAA-3^/^

### Statistical analysis

Each treatment consisted of three replicates (biological replicates) and each experiment was carried out twice (technical replicates) at different times. Results are mean of three replicates ± standard error. For statistical analysis of the data for significance, the paired two sample t-test was done with the help of Microsoft Excel 2010, which shows the significant variations between untreated control and post-imbibitional salinity stress-raised seedlings.

Hierarchical cluster analyses were performed to identify the close as well as distant relationship among the 10 experimental rice landraces of coastal area of Sundarban, Bangladesh by using IBM SPSS Statistics 20.

Among the parameters of germination and early growth performance, oxidative membrane damage and antioxidant (enzymatic & non-enzymatic) activity, a correlation study was made following the Pearson’s correlation method using IBM SPSS 2023.

## Supplementary Information

Supplementary Information.

## Data Availability

All data generated or analyzed during this study are included in this published article [and its supplementary information files].
