# Supplementary material for: Exploring the parameters of central redox hub for screening salinity tolerant rice landraces of coastal Bangladesh
Source: Sci Rep. 2022 Jul 29;12:12989. doi: 10.1038/s41598-022-17078-2 (PMC9338030; doi:10.1038/s41598-022-17078-2)

**Supplementary table 1:** **Pilot experiment for standardization of treatment conditions (Post imbibitional salinity stress) of seeds of ten experimental rice landraces based on dose – response results of the effect of different magnitudes of post imbibitional salinity stress on quality redox parameters [ROS (H_2_O_2_ ) accumulation, total radical scavenging property and protein oxidation (free carbonyl content)] of 168 hour old seedlings of ten experimental landraces of Sundarban , Bangladesh. Results are mean of three replicates ± standard error.**

| **Rice**  **landrace** | **Quality Redox Parameters under different treatment conditions** | | | | | | | | | | | | | | | | | |
| --- | --- | --- | --- | --- | --- | --- | --- | --- | --- | --- | --- | --- | --- | --- | --- | --- | --- | --- |
|  | **H_2_O_2_ accumulation**  **(m mol g^-1^dm)** | | | | | | **DPPH Redial Scavenging Property**  **( % radical scavenged g^-1^ dm)** | | | | | | **Free Carbonyl Content**  **(m mol g^-1^dm)** | | | | | |
|  | **Control** | **50mM**  **NaCl** | **100mM**  **NaCl** | **150mM**  **NaCl** | **200mM**  **NaCl** | **250mM**  **NaCl** | **Control** | **50mM**  **NaCl** | **100mM**  **NaCl** | **150mM NaCl** | **200mM NaCl** | **250mM NaCl** | **Control** | **50mM**  **NaCl** | **100mM NaCl** | **150mM NaCl** | **200mM NaCl** | **250mM NaCl** |
| **Benapol** | **0.65**  **±**  **0.014** | **0.87**  **±**  **0.007** | **0.89**  **±**  **0.015** | **1.05**  **±**  **0.004** | **1.85**  **±**  **0.025** | **2.67**  **±**  **0.006** | **89.41**  **±**  **0.017** | **89.52**  **±**  **0.072** | **89.67**  **±**  **0.008** | **89.88**  **±**  **0.004** | **90.88**  **±**  **0.030** | **86.54**  **±**  **0.027** | **20.67**  **±**  **0.018** | **21.47**  **±**  **0.003** | **26.69**  **±**  **0.035** | **40.21**  **±**  **0.002** | **46.37**  **±**  **0.005** | **72.89**  **±**  **0.003** |
| **Talmugur** | **0.77**  **±**  **0.056** | **0.82**  **±**  **0.002** | **0.95**  **±**  **0.004** | **1.10**  **±**  **0.007** | **1.82**  **±**  **0.035** | **2.56**  **±**  **0.003** | **92.5**  **±**  **0.089** | **92.80**  **±**  **0.005** | **93.12**  **±**  **0.230** | **93.36**  **±**  **0.015** | **93.91**  **±**  **0.004** | **91.04**  **±**  **0.003** | **32.14**  **±**  **0.004** | **39.40**  **±**  **0.040** | **41.66**  **±**  **0.015** | **48.49**  **±**  **0.003** | **58.46**  **±**  **0.014** | **71.52**  **±**  **0.002** |
| **Kajolshail** | **0.84**  **±**  **0.004** | **0.85**  **±**  **0.001** | **1.33**  **±**  **0.003** | **1.80**  **±**  **0.002** | **2.40**  **±**  **0.004** | **3.08**  **±**  **0.003** | **85.21**  **±**  **0.012** | **85.29**  **±**  **0.001** | **86.27**  **±**  **0.005** | **86.41**  **±**  **0.003** | **87.39**  **±**  **0.028** | **82.49**  **±**  **0.025** | **19.73**  **±**  **0.016** | **24.11**  **±0.037** | **28.73**  **±**  **0.002** | **42.78**  **±**  **0.018** | **60.83**  **±**  **0.025** | **88.84**  **±**  **0.035** |
| **Kutepatnai** | **0.75**  **±**  **0.002** | **0.75**  **±**  **0.003** | **0.81**  **±**  **0.025** | **0.97**  **±**  **0.153** | **1.76**  **±**  **0.007** | **2.07**  **±**  **0.003** | **94.67**  **±**  **0.015** | **95.07**  **±**  **0.028** | **96.05**  **±**  **0.027** | **96.18**  **±**  **0.035** | **96.66**  **±**  **0.045** | **93.12**  **±**  **0.005** | **41.18**  **±**  **0.004** | **43.3**  **±**  **0.002** | **47.19**  **±**  **0.032** | **55.67**  **±**  **0.022** | **60.62**  **±**  **0.002** | **70.79**  **±**  **0.003** |
| **Rajashail** | **0.69**  **±**  **0.046** | **0.72**  **±**  **0.002** | **0.83**  **±**  **0.003** | **1.78**  **±**  **0.013** | **2.41**  **±**  **0.025** | **3.24**  **±**  **0.007** | **90.27**  **±**  **0.014** | **90.59**  **±**  **0.018** | **91.37**  **±**  **0.007** | **91.50**  **±**  **0.051** | **91.95**  **±**  **0.072** | **87.54**  **±**  **0.362** | **21.81**  **±**  **0.002** | **24.32**  **±**  **0.065** | **29.08**  **±**  **0.004** | **44.46**  **±**  **0.003** | **52.82**  **±**  **0.035** | **90.43**  **±**  **0.003** |
| **Kachra** | **0.86**  **±**  **0.007** | **0.89**  **±**  **0.001** | **1.18**  **±**  **0.045** | **1.84**  **±**  **0.045** | **2.98**  **±**  **0.005** | **3.76**  **±**  **0.36** | **95.33**  **±**  **0.004** | **95.39**  **±**  **0.015** | **95.81**  **±**  **0.054** | **95.84**  **±**  **0.007** | **95.87**  **±**  **0.054** | **90.73**  **±**  **0.056** | **42.79**  **±**  **0.003** | **46.22**  **±**  **0.036** | **49.51**  **±**  **0.004** | **70.64**  **±**  **0.002** | **114.16**  **±**  **0.056** | **135.26**  **±**  **0.025** |
| **Nonakochi** | **0.96**  **±**  **0.015** | **0.98**  **±**  **0.002** | **1.11**  **±**  **0.018** | **1.42**  **±**  **0.007** | **2.02**  **±**  **0.003** | **2.64**  **±**  **0.004** | **91.16**  **±**  **0.08** | **91.54**  **±**  **0.003** | **91.78**  **±**  **0.023** | **92.82**  **±**  **0.003** | **93.13**  **±**  **0.067** | **90.46**  **±0.022** | **21.27**  **±**  **0.020** | **23.47**  **±**  **0.003** | **26.92**  **±**  **0.045** | **32.43**  **±**  **0.004** | **42.61**  **±**  **0.016** | **72.14**  **±**  **0.086** |
| **Charobalam** | **0.98**  **±**  **0.003** | **1.08**  **±**  **0.003** | **1.51**  **±**  **0.027** | **2.03**  **±**  **0.007** | **3.12**  **±**  **0.015** | **4.16**  **±**  **0.01** | **89.06**  **±**  **0.023** | **89.12**  **±**  **0.0231** | **89.68**  **±**  **0.005** | **89.75**  **±**  **0.024** | **89.91**  **±**  **0.002** | **85.42**  **±**  **0.001** | **27.62**  **±**  **0.015** | **30.54**  **±**  **0.012** | **36.66**  **±**  **0.015** | **45.11**  **±**  **0.001** | **99.52**  **±**  **0.015** | **133.22**  **±**  **0.067** |
| **Lalmota** | **0.93**  **±**  **0.002** | **1.14**  **±**  **0.002** | **1.37**  **±**  **0.055** | **1.89**  **±**  **0.093** | **2.98**  **±**  **0.024** | **3. 88**  **±**  **0.006** | **88.18**  **±**  **0.051** | **88.19**  **±**  **0.0141** | **88.29**  **±**  **0.009** | **88.57**  **±**  **0.005** | **88.99**  **±**  **0.029** | **85.69**  **±**  **0.035** | **23.57**  **±**  **0.007** | **29.64**  **±**  **0.015** | **42.08**  **±**  **0.002** | **52.81**  **±**  **0.002** | **77.75**  **±**  **0.004** | **98.24**  **±**  **0.002** |
| **Jotaibalam** | **0.79**  **±**  **0.016** | **0.82**  **±**  **0.002** | **1.16**  **±**  **0.007** | **1.91**  **±**  **0.002** | **2.76**  **±**  **0.016** | **3.59**  **±**  **0.003** | **91.08**  **±**  **0.021** | **91.16**  **±**  **0.005** | **91.23**  **±**  **0.012** | **91.55**  **±**  **0.015** | **91.81**  **±**  **0.018** | **89.85**  **±**  **0.08** | **31.21**  **±**  **0.024** | **37.44**  **±**  **0.035** | **48.15**  **±**  **0.026** | **84.54**  **±**  **0.004** | **101.50**  **±**  **0.002** | **127.49**  **±**  **0.016** |

**Supplementary table 2:** **Results of** **Pilot experiment for standardization of treatment conditions (Post imbibitional salinity stress) of seeds of ten experimental rice landraces based on dose – response results of the effect of different magnitudes of post imbibitional salinity stress on germination and early growth phenotypes (T_50_ value and relative growth index). Results are mean of three replicates ± standard error**

| **Rice**  **Landrace** | **Germination Performance under different treatment conditions** | | | | | | | | | | | |
| --- | --- | --- | --- | --- | --- | --- | --- | --- | --- | --- | --- | --- |
|  | **T_50_ value**  **(hrs)** | | | | | | **Relative Growth Index (RGI)**  **(%)** | | | | | |
|  | **Control** | **50mM**  **NaCl** | **100mM**  **NaCl** | **150mM**  **NaCl** | **200mM**  **NaCl** | **250mM**  **NaCl** | **Control** | **50mM**  **NaCl** | **100mM**  **NaCl** | **150mM**  **NaCl** | **200mM**  **NaCl** | **250mM**  **NaCl** |
| **Benapol** | **28.34±0.562** | **30.43±0.477** | **31.51±0.422** | **33.22±0.82** | **39.51±0.145** | **52.67±0.078** | **100±00** | **98.28±0.104** | **95.84±0.089** | **57.05±0.082** | **45.95±0.025** | **38.12±0.051** |
| **Talmugur** | **22.6±0.321** | **26.29±0.544** | **30.52±0.611** | **32.43±0.47** | **36.84±0.174** | **44.29±0.601** | **100±00** | **99.243±0.134** | **88.81±0.033** | **78.69±0.028** | **66.99±0.063** | **56.99±0.132** |
| **Kajolshail** | **23.68±0.226** | **24.48±0.079** | **26.34±0.223** | **35.51±0.026** | **43.53±0.425** | **55.67±0.024** | **100±00** | **98.05±0.237** | **67.74±0.057** | **46.23±0.053** | **34.15±0.176** | **30.04±0.092** |
| **Kutepatnai** | **20.51±0.301** | **21.48±0.1207** | **22.71±0.214** | **25.49±0.51** | **26.68±0.234** | **41.32±0.084** | **100±00** | **98.98±0.182** | **87.41±0.064** | **82.76±0.064** | **71.28±0.071** | **65.62±0.117** |
| **Rajashail** | **29.18±0.293** | **33.57±0.343** | **39.6±0.023** | **42.22±0.523** | **46.84±0.276** | **58.62±0.058** | **100±00** | **96.67±0.053** | **80.42±0.118** | **50.34±0.118** | **31.74±0.153** | **26.09±0.045** |
| **Kachra** | **22.67±0.158** | **28.68±0.285** | **32.20±0.105** | **44.67±0.088** | **54.18±0.373** | **65.84±0.223** | **100±00** | **86.75±0.103** | **64.22±0.015** | **38.96±0.048** | **28.14±0.057** | **23.07±0.108** |
| **Nonakochi** | **28.51±0.045** | **29.32±0.198** | **30.84±0.184** | **34.89±0.091** | **39.18±0.281** | **46.77±0.311** | **100±00** | **99.82±0.011** | **93.09±0.107** | **85.294±0.127** | **45.26±0.082** | **37.95±0.069** |
| **Charobalam** | **20.84±0.124** | **24.68±0.385** | **28.33±0.014** | **48.69±0.014** | **60.84±0.078** | **79.89±0.126** | **100±00** | **90.85±0.146** | **53.19±0.032** | **36.54±0.097** | **20.85±0.137** | **17.69±0.054** |
| **Lalmota** | **24.08±0.501** | **28.73±0.173** | **36.46±0.602** | **40.24±0.801** | **47.51±0.542** | **60.44±0.421** | **100±00** | **94.72±0.163** | **48.23±0.122** | **42.65±0.134** | **31.14±0.234** | **24.49±0.023** |
| **Jotaibalam** | **29.34±0.178** | **31.47±0.145** | **32.68±0.178** | **41.76±0.028** | **51.84±0.471** | **63.22±0.762** | **100±00** | **90.67±0.085** | **53.25±0.041** | **40.51±0.053** | **28.78±0.107** | **20.67±0.087** |

**Supplementary table 3: Effect of post imbibitional salinity stress (PISS- 200mM NaCl) on the ion contents and Na^+^/K^+^ ratio of 7-day- old rice seedlings of ten landraces. Results are mean of three replicates ± standard error. *Significant from control at 0.05 level (t-test). **Significant from control at 0.01 level (t-test).**

| Rice Landraces | Treatment | Na^+^ (m mol g^-1^dm) | K^+^ (m mol g^-1^dm) | Cl**^-^** (m mol g^-1^dm) | Na^+^/K^+^ |
| --- | --- | --- | --- | --- | --- |
| Benapol | Control | 0.0617 ± 0.0134 | 0.9259 ± 0.0376 | 0.2512 ± 0.0462 | 0.085 ± 0.0042 |
|  | Stress | 0.5766 ± 0.0679 | 0.4267 ± 0.0129* | 0.8217 ± 0.0350* | 1.2201 ± 0.0093** |
| Talmugur | Control | 0.1049 ± 0.0047 | 1.3761 ± 0.2293 | 0.2548 ± 0.0406 | 0.0762 ± 0.0135 |
|  | Stress | 0.795 ± 0.0943 | 0.8573 ± 0.0406 | 0.6679 ± 0.0082* | 0.9273 ± 0.0088** |
| Kajolshail | Control | 0.0811 ± 0.01 | 1.3003 ± 0.0717 | 0.1209 ± 0.0043 | 0.0624 ± 0.0150 |
|  | Stress | 0.8769 ± 0.117 | 0.5709 ± 0.0440* | 0.7084 ± 0.0462 | 1.4593±0.0279** |
| Kutepatnai | Control | 0.1036 ± 0.0116 | 1.3836 ± 0.0694 | 0.2428 ± 0.0082 | 0.0762 ± 0.0159 |
|  | Stress | 0.6583 ± 0.1671 | 0.8235 ± 0.0319* | 0.7011 ± 0.0120** | 0.7993 ± 0.0046** |
| Rajashail | Control | 0.1121 ± 0.0257 | 1.253 ±0.1248 | 0.3437 ± 0.0120 | 0.0895 ± 0.0214 |
|  | Stress | 0.7765 ± 0.0042* | 0.4746± 0.0300 | 0.8722 ± 0.0168** | 1.6181 ± 0.0279** |
| Kachra | Control | 0.1079 ± 0.0361 | 1.2687 ± 0.0457 | 0.3517 ± 0.0462 | 0.085 ± 0.0214 |
|  | Stress | 0.8105 ± 0.1261 | 0.3968 ± 0.0423* | 0.9866 ± 0.1215 | 2.0426 ± 0.1215* |
| Nonakochi | Control | 0.0516 ± 0.0161 | 1.1656 ± 0.0394 | 0.1267 ± 0.0043 | 0.0442 ± 0.0042 |
|  | Stress | 0.6983 ± 0.0900 | 0.6447 ± 0.0339* | 0.6282 ± 0.0406 | 1.0831 ± 0.0135** |
| Charobalam | Control | 0.0597 ± 0.0123 | 1.1921 ± 0.0139 | 0.2087 ± 0.0082 | 0.05 ± 0.0150 |
|  | Stress | 1.3406 ± 0.3239 | 0.4437 ± 0.0390* | 0.9166 ± 0.1215 | 3.0214 ± 0.1215* |
| Lalmota | Control | 0.0687 ± 0.0225 | 0.9633 ± 0.0462 | 0.1998 ± 0.0406 | 0.0713 ± 0.0042 |
|  | Stress | 0.6808 ± 0.1744 | 0.33 ± 0.0323* | 0.8825 ± 0.0462** | 2.063 ± 0.0279** |
| Jotaibalam | Control | 0.0565 ± 0.0102 | 1.1704 ± 0.2767 | 0.2306 ± 0.0043 | 0.0482 ± 0.0046 |
|  | Stress | 1.0413 ± 0.0281* | 0.529 ± 0.0093 | 0.8783 ± 0.0350* | 1.9684 ± 0.0279** |

**Supplementary table 4: Correlation among the parameters of germination & early growth performance, oxidative membrane damage and antioxidant (enzymatic & non- enzymatic) activity of the seedling of ten landraces of rice of Sundarban, Bangladesh raised from post-imbibitional salinity stress (PISS-200mM/0.18Scm^-1^ NaCl)**.

|  | **t_50_** | **Germination Rate** | **Vigor Index** | **Hydroperoxide** | **TBARS** | **C=O** | **Conjugated Diene** | **Lypoxigenase** | **H_2_O_2_** | **Catalase** | **Glutathione Reductase** | **Superoxide dismutase** | **Ascorbate peroxidase** | **Total Glutathione** | **Total Ascorbate** |
| --- | --- | --- | --- | --- | --- | --- | --- | --- | --- | --- | --- | --- | --- | --- | --- |
| **t_50_** | 1 | -.875** | -.504** | .896** | .601** | .835** | .617** | .842** | .926** | .426** | -0.246 | 0.008 | 0.128 | .691** | .527** |
| **Germination Rate** | -.875** | 1 | .452** | -.861** | -.719** | -.852** | -.639** | -.736** | -.873** | -.386** | 0.082 | -.292* | -0.25 | -.577** | -.452** |
| **Vigor Index** | -.504** | .452** | 1 | -.458** | -.269* | -.400** | -.392** | -.426** | -.501** | 0.022 | -0.003 | 0.037 | 0.214 | -0.221 | -0.109 |
| **Hydroperoxide** | .896** | -.861** | -.458** | 1 | .591** | .802** | .788** | .754** | .857** | 0.223 | -0.084 | 0.15 | 0.075 | .689** | .291* |
| **TBARS** | .601** | -.719** | -.269* | .591** | 1 | .551** | .370** | .365** | .567** | 0.007 | -.287* | .403** | .449** | 0.185 | 0.145 |
| **C=O** | .835** | -.852** | -.400** | .802** | .551** | 1 | .497** | .893** | .893** | .386** | 0.119 | 0.109 | 0.099 | .604** | .476** |
| **Conjugated Diene** | .617** | -.639** | -.392** | .788** | .370** | .497** | 1 | .335** | .570** | 0.034 | -0.096 | .385** | -0.029 | .460** | -0.04 |
| **Lypoxigenase** | .842** | -.736** | -.426** | .754** | .365** | .893** | .335** | 1 | .887** | .474** | 0.1 | -.276* | 0.033 | .664** | .624** |
| **H_2_O_2_** | .926** | -.873** | -.501** | .857** | .567** | .893** | .570** | .887** | 1 | .431** | -0.116 | 0.034 | 0.126 | .655** | .508** |
| **Catalase** | .426** | -.386** | 0.022 | 0.223 | 0.007 | .386** | 0.034 | .474** | .431** | 1 | -0.052 | -0.055 | .516** | .597** | .894** |
| **Glutathione Reductase** | -0.246 | 0.082 | -0.003 | -0.084 | -.287* | 0.119 | -0.096 | 0.1 | -0.116 | -0.052 | 1 | 0.024 | 0.002 | 0.102 | -0.085 |
| **Superoxide dismutase** | 0.008 | -.292* | 0.037 | 0.15 | .403** | 0.109 | .385** | -.276* | 0.034 | -0.055 | 0.024 | 1 | .349** | 0.073 | -0.213 |
| **Ascorbate peroxidase** | 0.128 | -0.25 | 0.214 | 0.075 | .449** | 0.099 | -0.029 | 0.033 | 0.126 | .516** | 0.002 | .349** | 1 | .278* | .403** |
| **Total Glutathione** | .691** | -.577** | -0.221 | .689** | 0.185 | .604** | .460** | .664** | .655** | .597** | 0.102 | 0.073 | .278* | 1 | .555** |
| **Total Ascorbate** | .527** | -.452** | -0.109 | .291* | 0.145 | .476** | -0.04 | .624** | .508** | .894** | -0.085 | -0.213 | .403** | .555** | 1 |
| ** Correlation is significant at the 0.01 level (2-tailed). | | | | | | | | | | | | | | | |
| * Correlation is significant at the 0.05 level (2-tailed). | | | | | | | | | | | | | | | |

**Supplementary figure 1: Germination and early growth phenotypes of landraces of rice (Benapol, Charobalam, Kutepatnai, Kachra and Kajolshail) exposed to post-imbibitional Salinity stress**


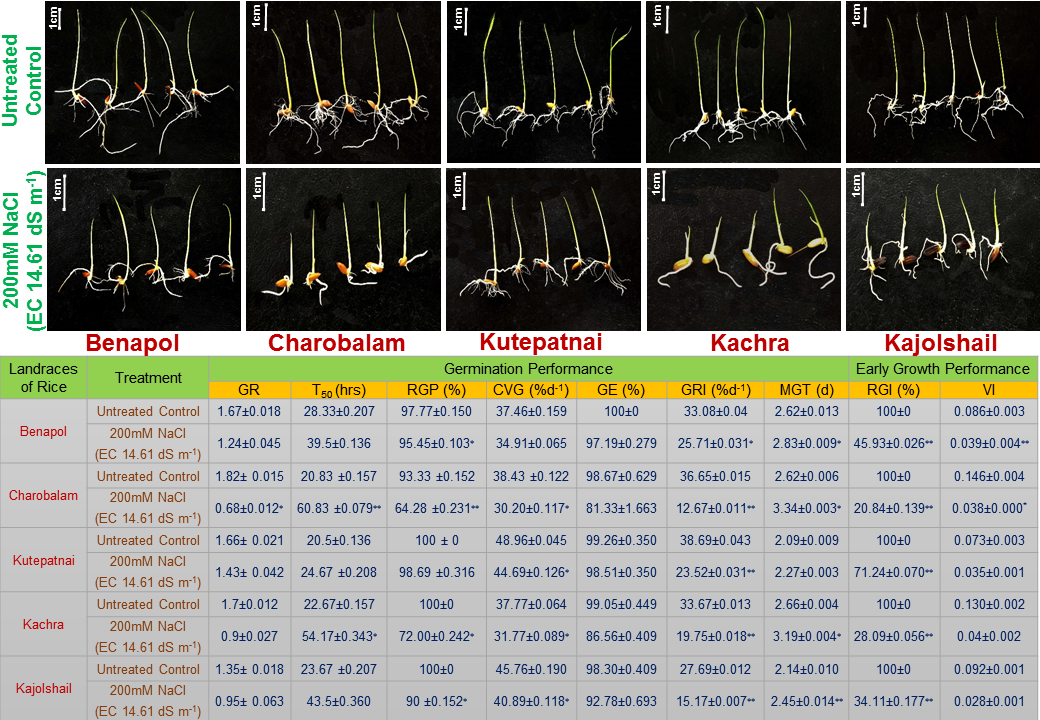


**Supplementary figure 2: Germination and early growth phenotypes of landraces of rice (Rajashail, Jotaibalam, Talmugur, Lalmota and Nonakochi) exposed to post-imbibitional Salinity stress**


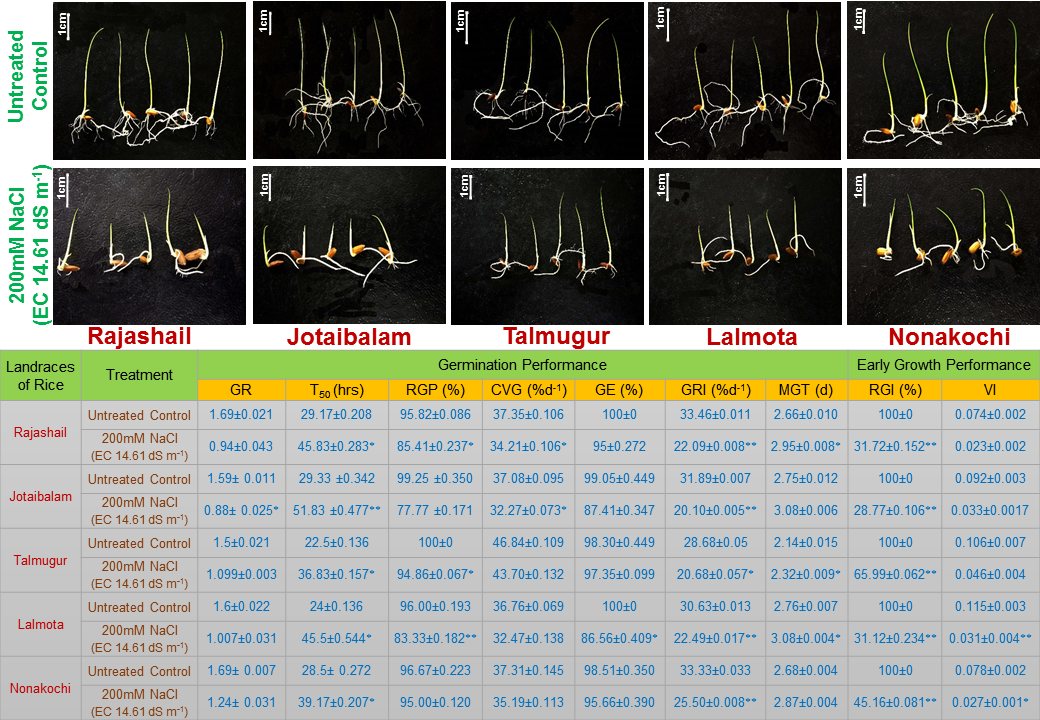


**Supplementary figure 3: a. Dendrogram of hierarchical cluster analysis depicting grouping in rice landraces based on the germination and early growth performances in the stress induced conditions (PISS)**

**b. Dendrogram of hierarchical cluster analysis depicting grouping in rice landraces based on the redox biomarkers in the stress induced conditions (PISS)**


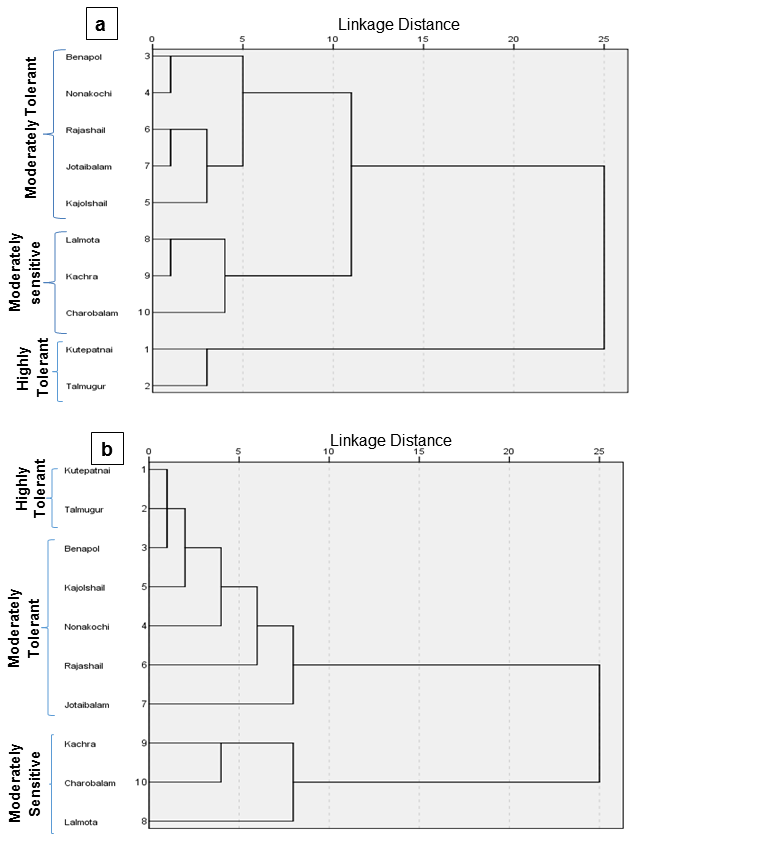


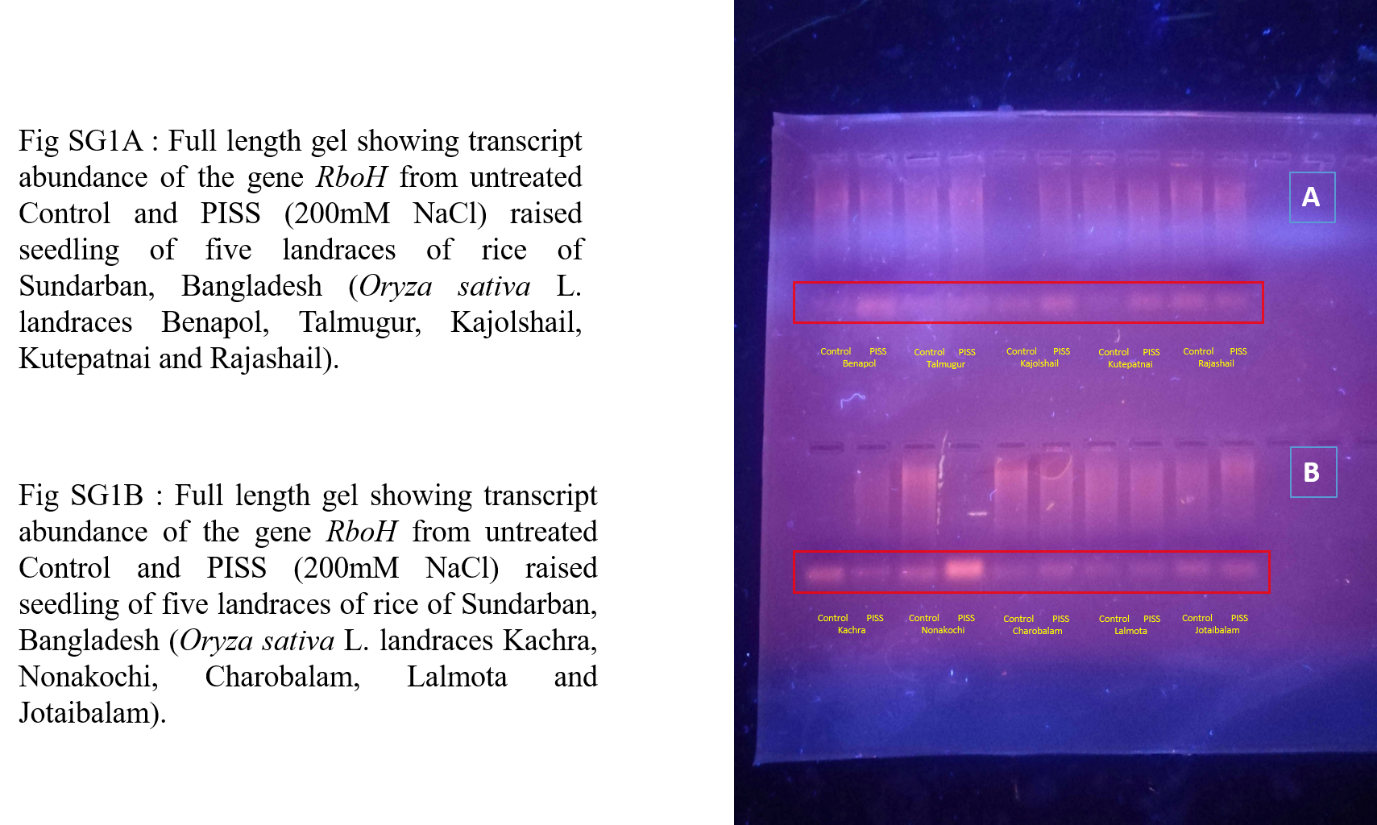
Original Gel Images


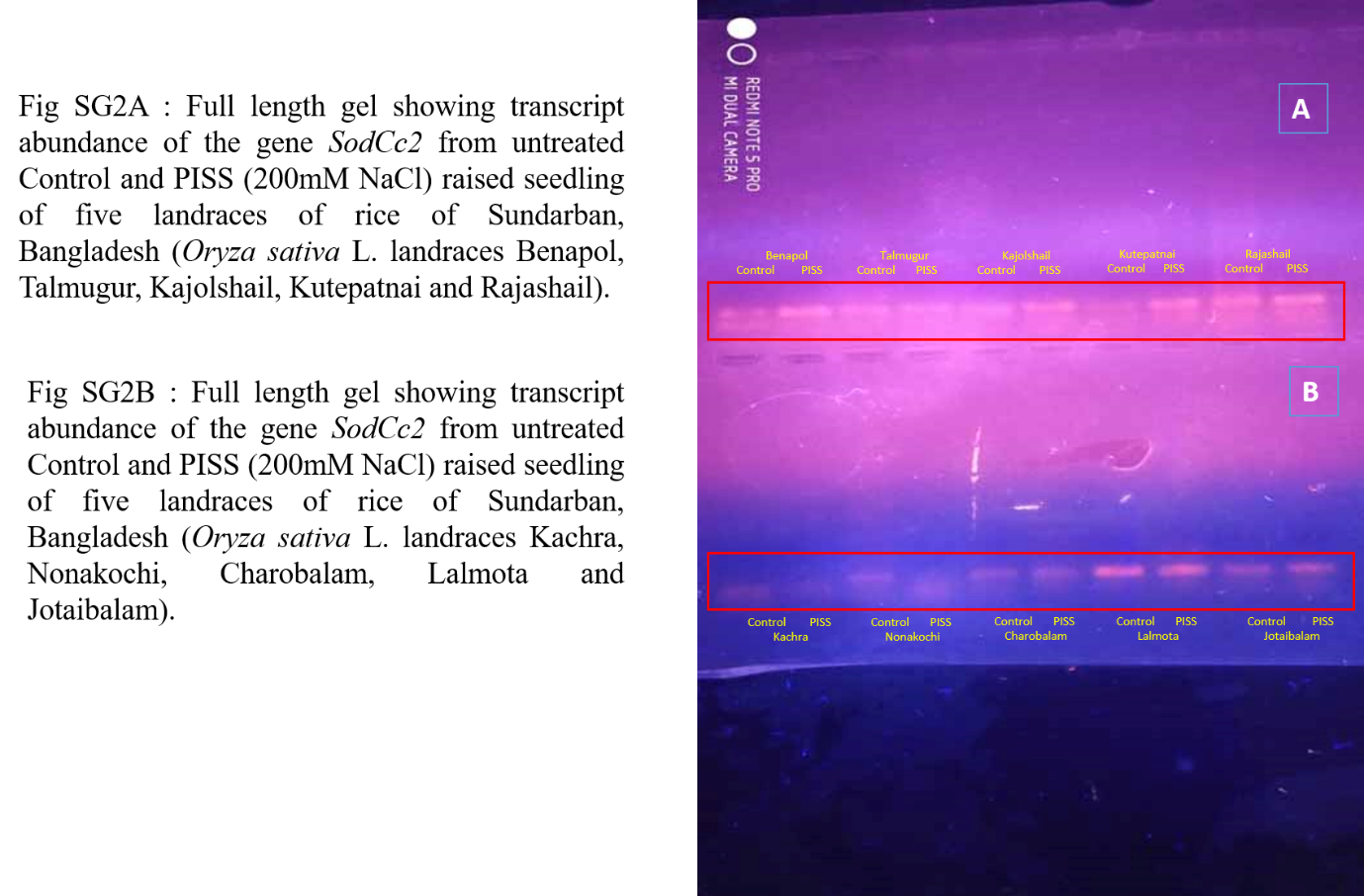


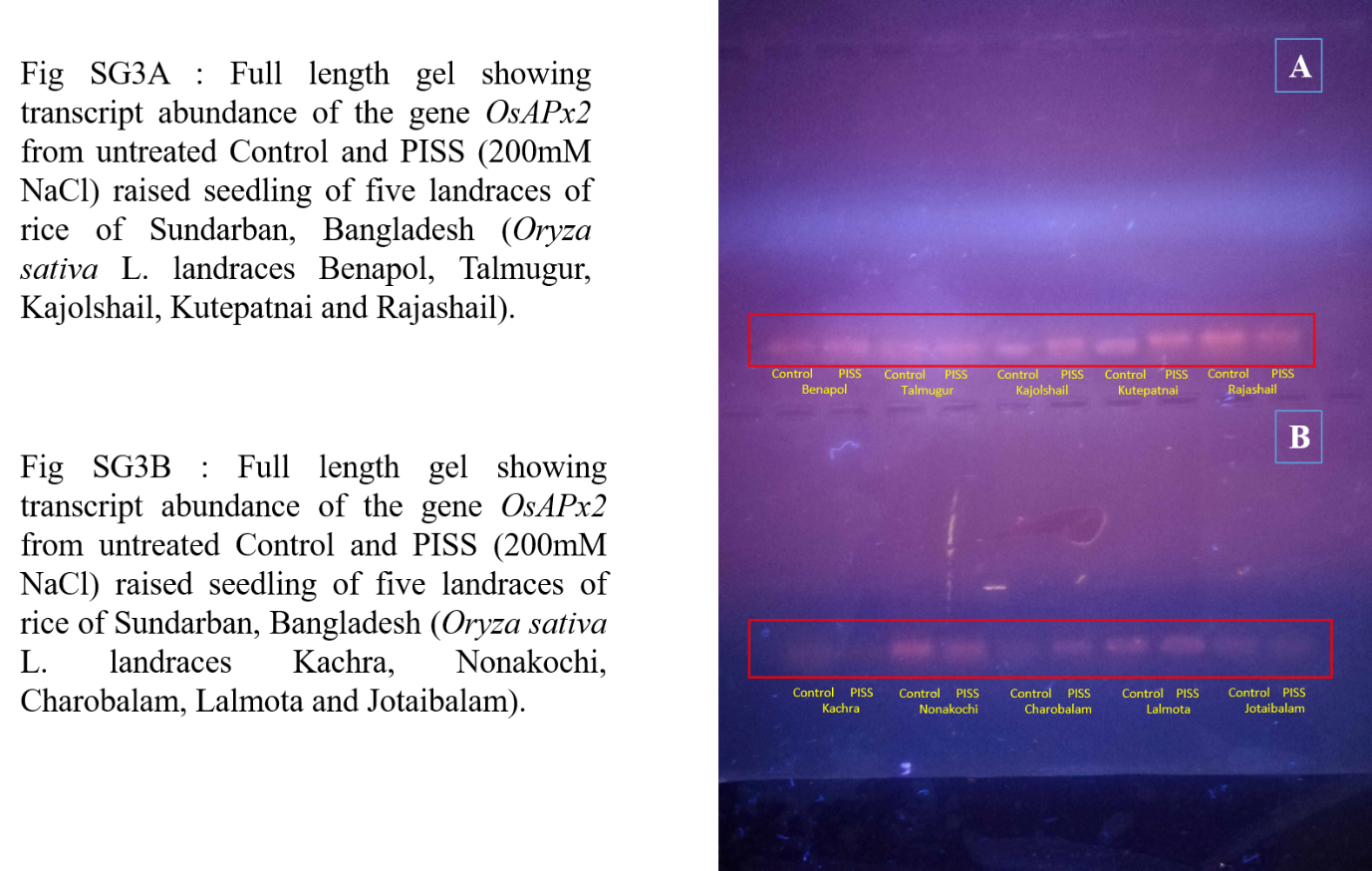


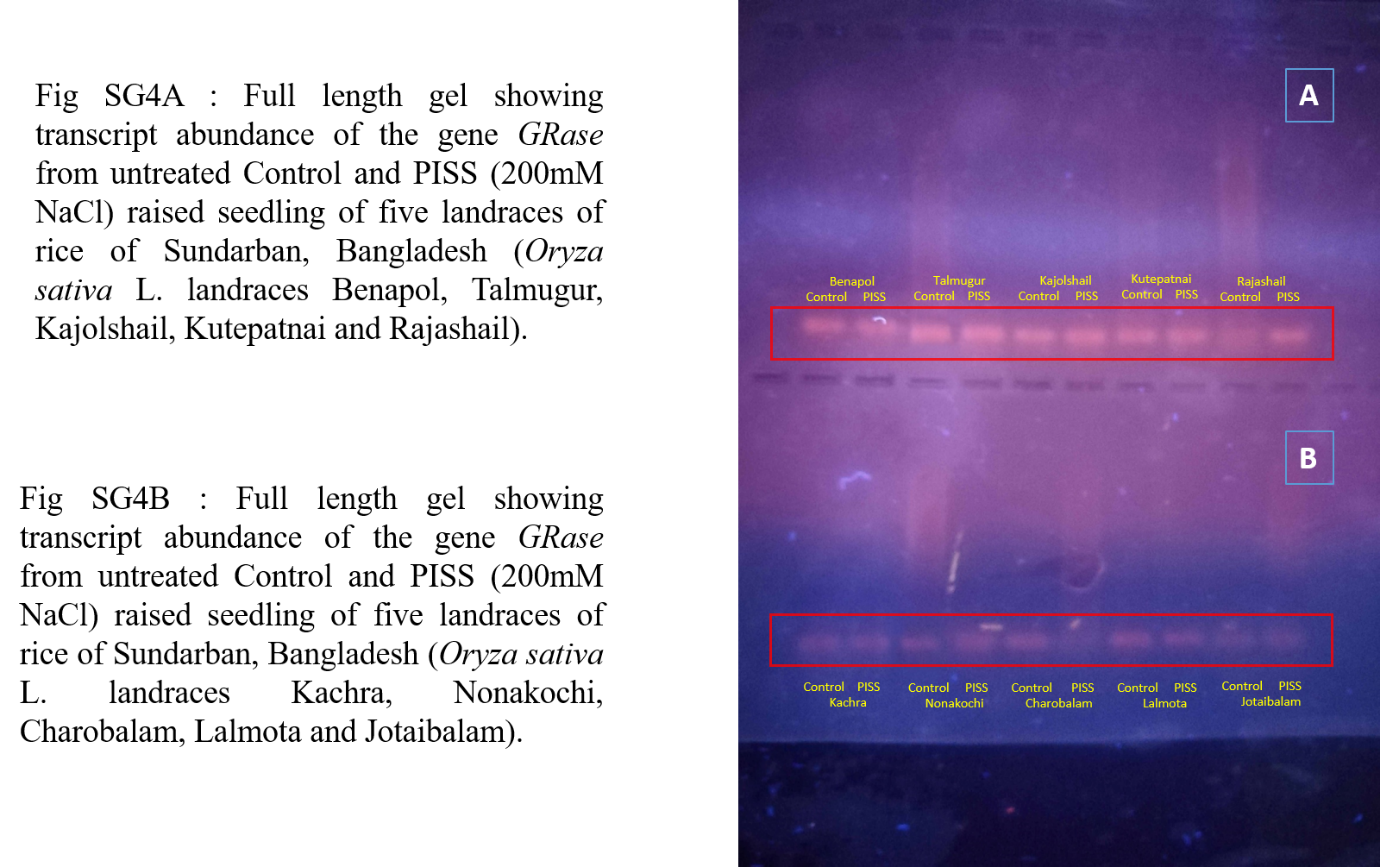


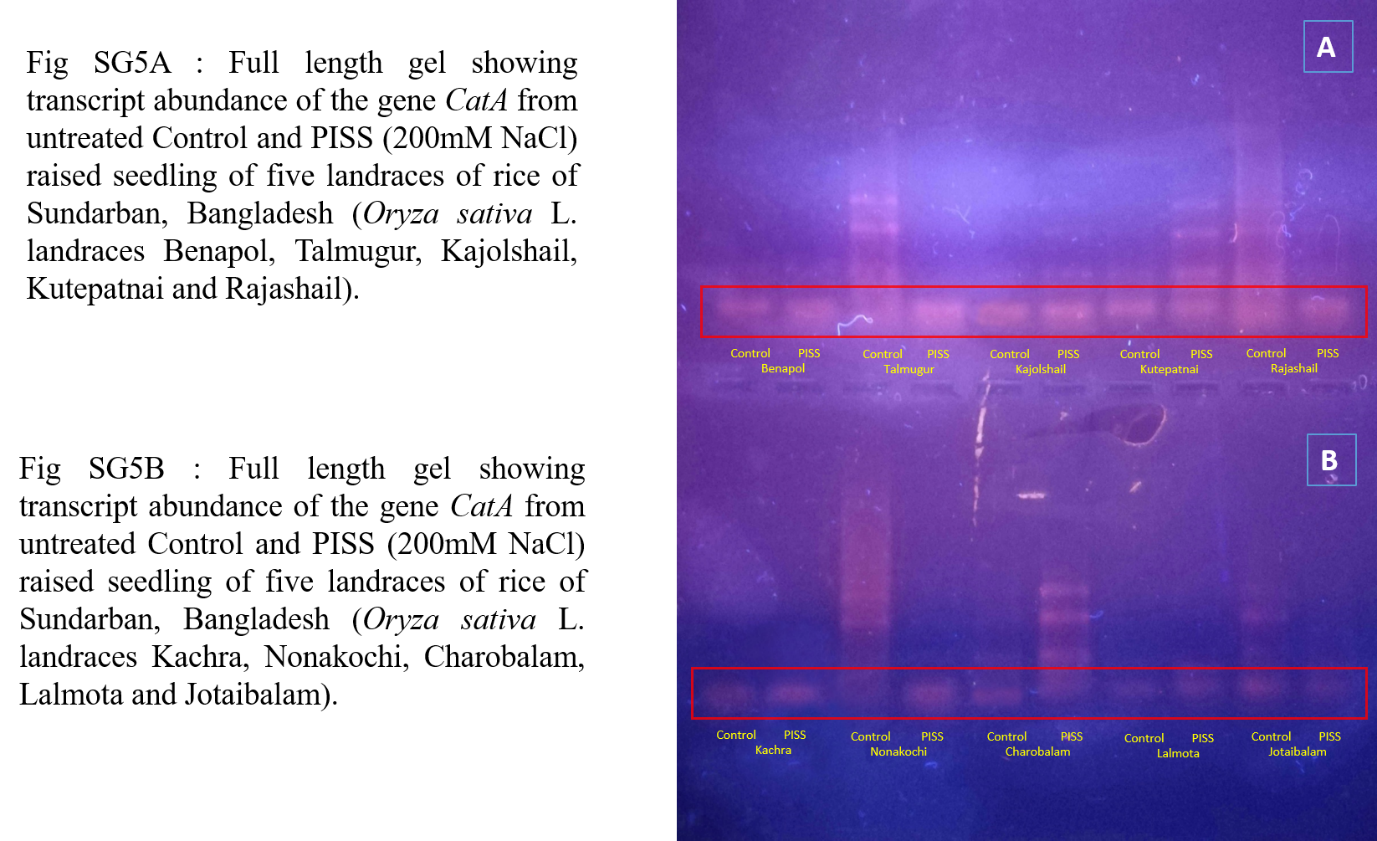


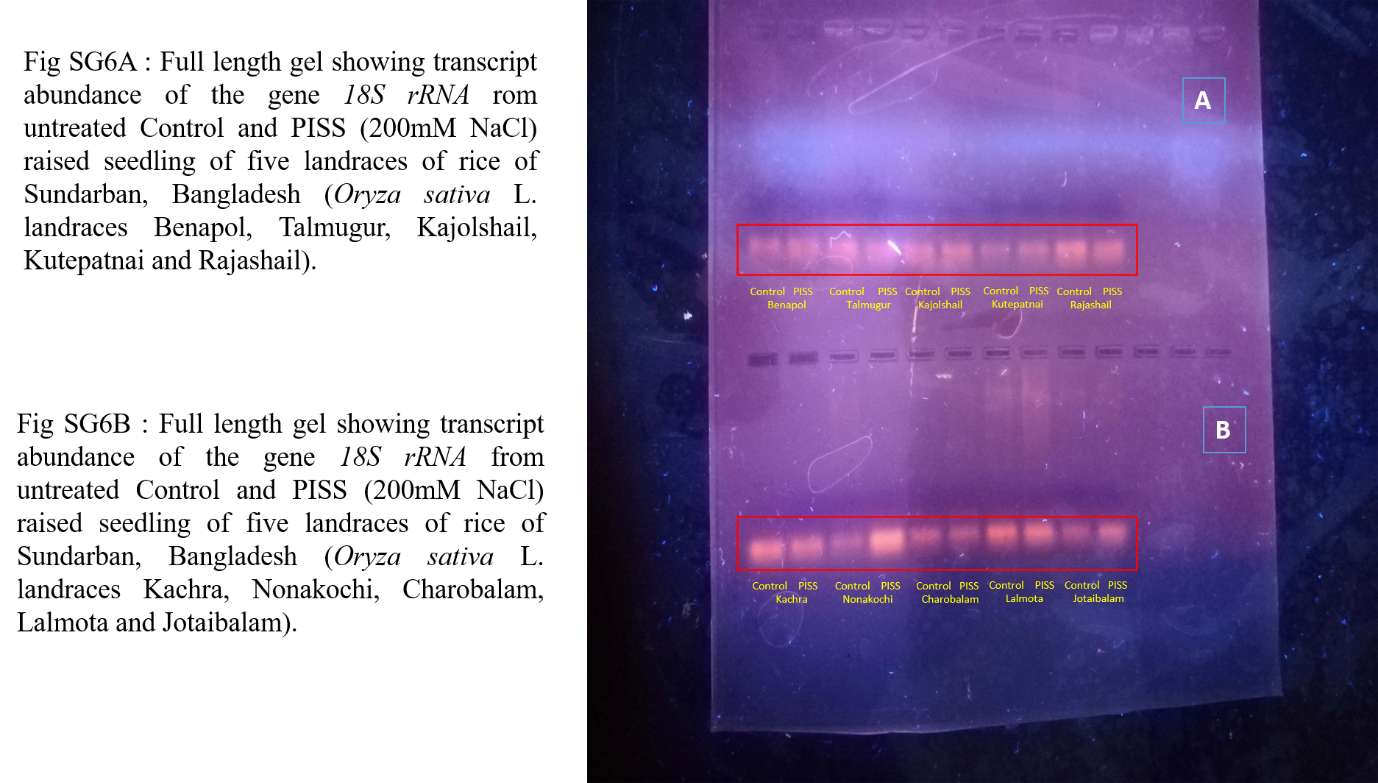

Supplement: Supplementary file 1 — Supplementary Information. [file 41598_2022_17078_MOESM1_ESM.docx]
